# Polysaccharide utilization loci and nutritional specialization in a dominant group of butyrate-producing human colonic *Firmicutes*

**DOI:** 10.1099/mgen.0.000043

**Published:** 2016-02-09

**Authors:** Paul O. Sheridan, Jennifer C. Martin, Trevor D. Lawley, Hilary P. Browne, Hugh M. B. Harris, Annick Bernalier-Donadille, Sylvia H. Duncan, Paul W. O'Toole, Karen P. Scott, Harry J. Flint

**Affiliations:** ^1^​ Rowett Institute of Nutrition and Health, University of Aberdeen, Bucksburn, Aberdeen AB21 9SB, UK; ^2^​ Wellcome Trust Sanger Institute, Hinxton CB10 1SA, UK; ^3^​ Department of Microbiology & Alimentary Pharmabiotic Centre, University College Cork, Cork, Ireland; ^4^​ Unité de Microbiologie INRA, Centre de Recherche de Clermont-Ferrand/Theix, 63122 Saint Genès Champanelle, France

**Keywords:** Carbohydrate, comparative genomics, gut microbiota, *Lachnospiraceae*, obligate anaerobe, *Roseburia*

## Abstract

*Firmicutes* and *Bacteroidetes* are the predominant bacterial phyla colonizing the healthy human large intestine. Whilst both ferment dietary fibre, genes responsible for this important activity have been analysed only in the *Bacteroidetes*, with very little known about the *Firmicutes*. This work investigates the carbohydrate-active enzymes (CAZymes) in a group of *Firmicutes*, *Roseburia* spp. and *Eubacterium rectale*, which play an important role in producing butyrate from dietary carbohydrates and in health maintenance. Genome sequences of 11 strains representing *E. rectale* and four *Roseburia* spp. were analysed for carbohydrate-active genes. Following assembly into a pan-genome, core, variable and unique genes were identified. The 1840 CAZyme genes identified in the pan-genome were assigned to 538 orthologous groups, of which only 26 were present in all strains, indicating considerable inter-strain variability. This analysis was used to categorize the 11 strains into four carbohydrate utilization ecotypes (CUEs), which were shown to correspond to utilization of different carbohydrates for growth. Many glycoside hydrolase genes were found linked to genes encoding oligosaccharide transporters and regulatory elements in the genomes of *Roseburia* spp. and *E. rectale*, forming distinct polysaccharide utilization loci (PULs). Whilst PULs are also a common feature in *Bacteroidetes*, key differences were noted in these *Firmicutes*, including the absence of close homologues of *Bacteroides* polysaccharide utilization genes, hence we refer to Gram-positive PULs (gpPULs). Most CAZyme genes in the *Roseburia*/*E. rectale* group are organized into gpPULs. Variation in gpPULs can explain the high degree of nutritional specialization at the species level within this group.

## Data Summary

The high-quality draft genomes generated in this work were deposited at the European Nucleotide Archive under the following accession numbers:

1. *Eubacterium rectale* T1-815; CVRQ01000001–CVRQ01000090: http://www.ebi.ac.uk/ena/data/view/PRJEB9320

2. *Roseburia faecis* M72/1; CVRR01000001–CVRR01000101: http://www.ebi.ac.uk/ena/data/view/PRJEB9321

3. *Roseburia inulinivorans* L1-83; CVRS01000001–CVRS01000151: http://www.ebi.ac.uk/ena/data/view/PRJEB9322

## Impact Statement

*Firmicutes* and *Bacteroidetes* are the predominant bacterial phyla that colonize the healthy human large intestine. Whilst both phyla include species that ferment dietary fibre, genes responsible for this important activity have been analysed only in the *Bacteroidetes* and this paper represents the first detailed analysis for a group of human colonic *Firmicutes*. This paper will be of interest to those working in the fields of bacterial genomics, intestinal microbiology, human nutrition and health, and microbial polysaccharide breakdown. In particular, interest is growing rapidly in the human gut microbiota and its contribution to health and disease, including the potential for manipulating the microbiota through diet to achieve health benefits. The bacteria studied here are of special interest as they play a dominant role in producing the health-protective metabolite butyrate from dietary carbohydrates. This analysis reveals distinct polysaccharide utilization loci that comprise genes encoding degradative enzymes (glycoside hydrolases (GHs)) linked to genes encoding carbohydrate transporters and regulatory functions in the genomes of *Roseburia* spp. and *Eubacterium rectale*. Key differences are reported between these PULs and those of colonic *Bacteroidetes*, whilst the GH distribution allows strains to be categorized into carbohydrate-utilization ecotypes that utilize different carbohydrates for growth.

## Introduction

The human large intestine supports an extremely dense and diverse microbial community that plays an important role in human health (Flint *et al.*, 2012b; Sekirov *et al.*, 2010). Carbohydrates derived from the diet and from the host that remain undigested by host enzymes provide the major energy sources for growth and metabolism of the colonic microbiota. In addition to interactions with the host involving microbial cells and cell components, the short-chain fatty acid products of carbohydrate fermentation by gut bacteria exert multiple effects on the host as energy sources, and as regulators of inflammation, proliferation and apoptosis (Louis *et al.*, 2014). There is particular interest in the role played by butyrate-producing species of the gut microbiota in health maintenance, as their populations are found to be less abundant in a range of conditions that involve dysbiosis, including inflammatory bowel disease and colorectal cancer (Balamurugan *et al.*, 2008; Wang *et al.*, 2012; Machiels *et al.*, 2014). The predominant butyrate-producing bacteria in the healthy human colon belong to the phylum *Firmicutes* (Barcenilla *et al.*, 2000; Louis *et al.*, 2010), and include *Faecalibacterium prausnitzii* (*Ruminococcaceae*) and *Roseburia* spp., *Eubacterium rectale*, *Eubacterium hallii* and *Anaerostipes* spp. (*Lachnospiraceae*) (Louis & Flint, 2009).

So far, the only group of human colonic bacteria to have been investigated in any detail with respect to polysaccharide utilization are *Bacteroides* spp. (Martens *et al.*, 2011; Flint *et al.*, 2012a). These species possess large genomes with extremely high numbers of predicted carbohydrate-active enzymes (CAZymes). These CAZyme genes are located in the genome adjacent to genes encoding regulators and carbohydrate transport functions, forming multiple polysaccharide utilization loci (PULs) whose organization is typified by the *Bacteroides thetaiotaomicron* starch utilization system (Sus) (Martens *et al.*, 2011; McNulty *et al.*, 2013; El Kaoutari *et al.*, 2013). This, together with the far lower proportional numbers of CAZymes found in the genomes of human colonic *Firmicutes*, has led to the suggestion that *Bacteroides* spp. play the predominant role in carbohydrate degradation in the human colon (El Kaoutari *et al.*, 2013). However, various *Firmicutes* have been shown to respond to changes in the major dietary carbohydrate in human volunteer studies, with relatives of *Ruminococcus* spp., *Roseburia* spp. and *E. rectale* increasing with diets enriched with resistant starch or wheat bran (Duncan *et al.*, 2007; Martínez *et al.*, 2010, 2013; Walker *et al.*, 2011; Salonen *et al.*, 2014; David *et al.*, 2014). This suggests an alternative interpretation, i.e. that *Firmicutes* might typically be nutritionally highly specialized, whereas *Bacteroides* spp. may typically retain a greater plasticity for glycan utilization. Such nutritional specialization has already been noted among the ruminococci (Ze *et al.*, 2012; Wegmann *et al.*, 2014). Given that *Firmicutes* can account for ∼70 % of bacterial phylogenetic diversity in the human colon (Eckburg *et al.*, 2005), there is an obvious need for better understanding of carbohydrate utilization in this phylum. *Roseburia* spp. together with *E. rectale* form a coherent group of butyrate-producing *Firmicutes*, based on 16S rRNA gene sequences and multiple shared genotypic and phenotypic traits, including butyrate pathway genes and flagellar motility (Aminov *et al.*, 2006; Louis & Flint, 2009; Neville *et al.*, 2013). The fact that this group of bacteria are flagellated provides an additional mechanism for interaction with the host immune system (Neville *et al.*, 2013). The availability of genome sequence information for multiple representatives of the *Roseburia* and *E. rectale* group isolated from the human colon therefore provides an excellent opportunity to gain an understanding of polysaccharide utilization by this important group of butyrate-producing *Firmicutes*, which typically accounts for 5–20 % of total colonic bacteria in human adults (Hold *et al.*, 2003; Aminov *et al.*, 2006; Tap *et al.*, 2009; Walker *et al.*, 2011). Our analysis reveals for the first time the existence and organization of Gram-positive PULs (gpPULs) in this group of *Lachnospiraceae*. Furthermore, considerable specialization in the utilization of different dietary carbohydrates was observed at the species level that is likely to underlie species-specific responses to dietary carbohydrates observed in human volunteer studies (Salonen *et al.*, 2014).

## Methods

### Genomes, bacterial strains and growth conditions

The bacterial genomes used in this work are described in Table 1. Routine culturing of bacterial strains was in anaerobic M2GSC medium (Miyazaki *et al.*, 1997) in 7.5 ml aliquots in Hungate tubes, sealed with butyl rubber septa (Bellco Glass). Single-carbohydrate growth experiments were carried out in basal YCFA medium (Lopez-Siles *et al.*, 2012) supplemented with 0.5 % (w/v) of the carbohydrate being examined. All carbohydrates and manufacturers are detailed in Table S1 (available in the online Supplementary Material). Cultures were inoculated using the anaerobic methods described by Bryant (1972) and incubated anaerobically without agitation at 37 °C. Growth experiments were routinely carried out in flat-bottom 96-well microtitre plates (Corning; Sigma-Aldrich) prepared in the anaerobic ConceptPlus workstation. Sample blanks containing uninoculated medium were used as controls. Substrates (10 μl of a 10 % stock) were placed directly in wells and a 190 μl aliquot of the master mix (7.5 ml basal YCFA containing 100 μl bacterial inoculum) was added. Microtitre plates were covered and tightly sealed (Bio-Rad iCycler iQ optical tape 2239444) to prevent evaporation and maintain the anaerobic atmosphere. Cells were incubated for 24 h at 37 °C in a BioTek spectrophotometer, with OD_650_ readings taken automatically every hour with low-speed shaking for 5 s prior to each reading. In cases where the substrate was particularly cloudy, experiments were repeated in basal YCFA (7.5 ml) Hungate tubes, containing 1 % (mucin T2 and T3), 0.5 % (inulin) or 0.2 % (β-mannan) substrate, and 100 μl inoculum.

**Table 1:** Genomes and bacterial strains All strains were isolated from human faecal samples. Sequencing and genome assembly of *E. rectale* T1-815, *R. inulinivorans* L1-83 and *R. faecis* M72/1 was performed by the Wellcome Trust Sanger Institute. The genomes of *E. rectale* A1-86, and M104/1 and *R. intestinalis* XB6B4 and M50/1 were sequenced by the Pathogen Genomics group at the Wellcome Trust Sanger Institute as part of the EU MetaHit project (http://www.sanger.ac.uk/resources/downloads/bacteria/metahit/). The locus tag is the strain identifier used throughout this work.

Species	Strain	Locus tag	GenBank accession No.	No. ORFs	Size (nt)	Genome publication reference	Strain publication reference	Institute of isolation^*^
*E. rectale*	ATCC33656	EUBREC_	NC_012781.1	3621	3 449 685	Unpublished	Unpublished	VPI
	A1-86	EUR_	NC_021010.1	2898	3 344 951	Unpublished	Barcenilla *et al.* (2000)	RINH
	M104/1	ERE_	NC_021044.1	3206	3 698 419	Unpublished	Louis *et al.* (2004)	RINH
	T1-815	T1_815_	CVRQ01000001–CVRQ01000090	2896	3 045 135	This work	Barcenilla *et al.* (2000)	RINH
*R. inulinivorans*	A2-194	RINU_	ACFY01000000	4522	4 048 462	Unpublished	Duncan *et al.* (2006)	RINH
	L1-83	L1-83_	CVRS01000001–CVRS01000151	3488	3 781 521	This work	Barcenilla *et al.* (2000)	RINH
*R. intestinalis*	L1-82	RINT_	ABYJ00000000.2	4766	4 411 375	Unpublished	Duncan *et al.* (2006)	RINH
	M50/1	ROI_	NC_021040.1	3461	4 143 550	Unpublished	Louis *et al.* (2004)	RINH
	XB6B4	RO1_	NC_021012	3610	4 286 292	Unpublished	Chassard *et al.* (2007)	INRA
*R. hominis*	A2-183	RHOM_	CP003040.1	3362	3 592 125	Travis *et al.* (2015)	Duncan *et al.* (2006)	RINH
*R. faecis*	M72/1	M72_	CVRR01000001–CVRR01000101	3205	3 334 694	This work	Duncan *et al.* (2006)	RINH

* VPI, Virginia Polytechnic Institute and State University, Blacksburg, VA, USA; RINH, Rowett Institute of Nutrition and Health, University of Aberdeen, Aberdeen, UK; INRA, INRA Clermont-Ferrand/Theix, France.

Gas production was measured by displacement of a syringe inserted into the butyl stopper following 48 h growth in Hungate tubes. The final pH of the media was recorded and compared with that of the starting medium. These formed additional checks to assess bacterial growth on cloudy substrates.

Substrate-agarose overlay plates were used to assess the ability of strains to degrade substrates, without necessarily being able to grow on them. Bacterial broth cultures grown overnight in Hungate tubes (M2GSC) were streaked onto YCFA agar plates containing 0.2 % glucose, soluble potato starch and cellobiose. Following overnight incubation, a hand-hot molten 0.4 % agarose overlay containing 0.2 % of the appropriate substrate prepared in 50 mM sodium phosphate buffer (pH 6.5) was carefully poured over the colonies. After a further overnight incubation, plates were stained for 30 min, washed and the formation of clear zones noted. Mucin overlays were stained with 0.1 % Amido black prepared in 3.5 M acetic acid and washed in 1.2 M acetic acid; glucagel overlays were stained with 0.1 % Congo red and washed with 1 M NaCl; β-mannan overlays were stained with either 0.1 % Congo red or with Grams Iodine Solution (Sigma).

### Sequencing, assembly and automated annotation of high-quality draft genomes of *E. rectale* T1-815, *Roseburia faecis* M72/1 and *Roseburia inulinivorans* L1-83

Genomic DNA was sequenced on the Illumina HiSeq platform generating paired-end reads with a read length of 100 bp. A *de novo* assembly of the three strains was carried out using Velvet (Zerbino & Birney, 2008), and the assemblies were manually improved using a combination of Gapfiller (Boetzer & Pirovano, 2012) to close sequence gaps and iCORN (Otto *et al.*, 2010) to correct for sequence errors. Annotation of the improved assemblies consisted of identifying coding sequences using Prodigal (Hyatt *et al.*, 2010) and transferring functional gene annotation using closely related references in a best-hit reciprocal manner. Further annotation was then incorporated, principally using Pfam (Punta *et al.*, 2012), Prosite (Sigrist *et al.*, 2010) and RNAmmer (Lagesen *et al.*, 2007) to identify protein families, functional protein sites and rRNA. These high-quality draft genomes were deposited at the European Nucleotide Archive.

### Pan-genome homology and motif identification

Orthology detection was performed using QuartetS software (Yu *et al.*, 2011). Orthologues were assigned based on the bidirectional best hit of amino acid sequences, with thresholds of 45 % sequence identity over 50 % of sequence. Additional criteria for orthologue prediction were *E* values < 1e^− 5^ and bit scores >50, and a minimum clustering number of two sequences. Sequences were then separated into the *Roseburia*/*E. rectale* group core and variable genome using a presence/absence matrix. Sequences with no orthologues in the other 10 strains were considered to be unique genes.

All protein sequences annotated as having an Enzyme Commission number of EC 3.2.1 [glycoside hydrolase (GH)] were extracted from the KEGG database (Kanehisa & Goto, 2000) to form a 24 981 amino acid sequence GH protein reference database. The proteins of the pan-genome of the *Roseburia*/*E. rectale* group were queried against this GH protein database using blastp. The results were filtered to exclude all matches with *E* values >1e^− 10^, sequence identity < 35 % or bit scores < 200. The database for carbohydrate-active enzyme annotation (dbCAN) HMM (hidden Markov model) database version 3 (http://csbl.bmb.uga.edu/dbCAN/) was downloaded locally and used to query the pan-genome for conserved domains with the programme hmmscan (a command in the hmmer 3.0 package; hmmer.org). These results were filtered by excluding *E* values >1e^− 3^ for alignments < 80 amino acids and *E* values >1e^− 5^ for alignments ≥ 80 amino acids, and using alignment coverage of >0.3 as the threshold.

### Carbohydrate utilization ecotype (CUE) and gpPUL determination

Where all the members of a GH family hydrolysed the same carbohydrate type, carbohydrate sets were assigned by GH family e.g. all GH13s were assigned to the α-glucans set. Where different members of a GH family hydrolysed different carbohydrate types, carbohydrate sets were assigned by KEGG GH annotation (Table S2). As type 1 arabinogalactan could be interpreted as belonging to the carbohydrate sets ‘Xylans and Arabinans’, ‘Pectins’ or ‘Alpha- and Beta-galactosides’, endo-1,4-β-galactanases, which cleave the 1,4-β-galactan backbone of type 1 arabinogalactan, were assigned to a separate carbohydrate set termed ‘Type-1 Arabinogalactans’. GH heatmap analyses were performed using MeV software from the tm4 suite (Saeed *et al.*, 2003). CUEs were determined by hierarchical clustering of the GH heatmap using Kendall τ distance and Spearman distance with complete linkage.

The pan-genome was queried against the KEGG and COG (http://www.ncbi.nlm.nih.gov/COG/) databases, excluding top hit matches of *E* values >1e^–5^. The putative CAZymes discovered in this work were incorporated into this annotation and GHs less than 11 genes from a putative carbohydrate transporter system were further investigated by manual curation in Artemis (Carver *et al.*, 2008). The boundaries of gpPULs, both upstream and downstream, were determined by the presence of three adjacent genes not predicted to be involved in carbohydrate degradation. gpPULs were defined as encoding, at minimum, a polysaccharide-degrading enzyme, a transport system and a transcriptional regulator.

### Additional annotation tools

Phylogenetic trees were reconstructed in mega 6 software (Kumar *et al.*, 2008). Principal coordinate analyses were performed in the statistical software r using Kendall τ distances of the first five eigenvectors. Visual comparisons of intra-species and inter-species genome variability were performed using the blast Ring Image Generator (brig) software (Alikhan *et al.*, 2011).

## Results

### *In vitro* utilization of carbohydrates by the *Roseburia*/*E. rectale* group

**Fig. 1.**
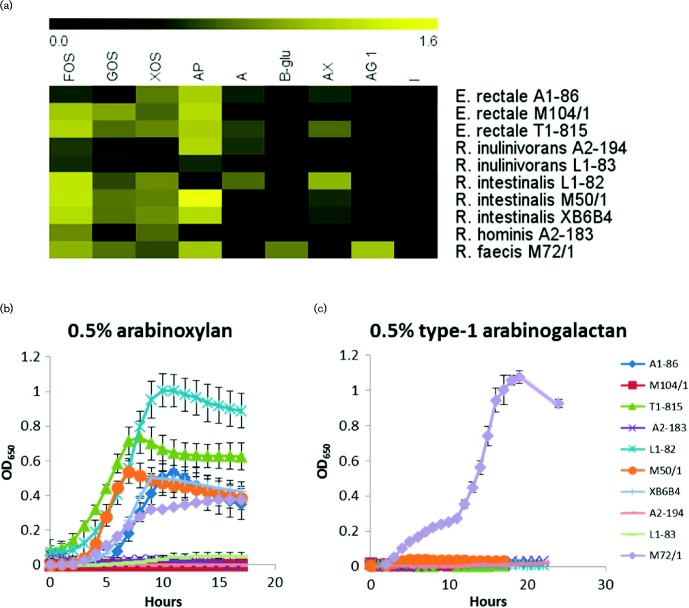
Growth of *Roseburia*/*E. rectale* strains on selected carbohydrates in microtitre plates. (a) Heatmap representing the mean maximum OD_650_ obtained by a strain during growth on a specific carbohydrate (OD_650_ 0.0–1.6). Growth was observed on fructo-oligosaccharide (FOS), galacto-oligosaccharide (GOS), xylo-oligosaccharide (XOS), amylopectin (AP), amylose (A), 1,3–1,4-β-glucan (B-glu), arabinoxylan (AX), type 1 arabinogalactan (AG1) and inulin (I). No growth was observed for any the 11 strains on β-mannan, xyloglucan, type 2 arabinogalactan, mucin core type 2 or mucin core type 3. Data plotted in graphs are the mean ± sd OD_650_ readings of six replicates of strains grown on (b) 0.5 % arabinoxylan or (c) 0.5 % type 1 arabinogalactan. Full growth data are presented in Table S3.

The ability of three strains of *E. rectale*, two of *R. inulinivorans*, three of *Roseburia intestinalis* and one each of *R. faecis* and *Roseburia hominis* to degrade and utilize a variety of carbohydrates for growth was tested by anaerobic culturing. Growth in microtitre plates revealed that all 10 strains could utilize fructo-oligosaccharides (Fig. 1a). The ability to grow on galacto-oligosaccharides and xylo-oligosaccharides was more limited with *E. rectale* A1-86, *R. inulinivorans* L1-83 and *R. hominis* A2-183 unable to utilize galacto-oligosaccharides, and the two *R. inulinivorans* strains unable to grow on xylo-oligosaccharides.

Nine of the 10 strains were able to utilize amylopectin and/or amylose for growth (Fig. 1a, Table S3), the exception being *R. hominis* A2-183 which was not unable to grow on either type of starch. All strains of *E. rectale* and *R. inulinivorans* were capable of utilizing inulin, whereas *R. intestinalis*, *R. hominis* and *R*. *faecis* strains did not grow with inulin as the sole carbohydrate source (Fig. S1).

Growth on the plant cell wall polysaccharides arabinoxylan, xyloglucan, arabinogalactan, 1,3–1,4-β-glucan and β-mannan was also variable. Arabinoxylan could be utilized by all the three *R. intestinalis* strains, by *E. rectale* A1-86 and T1-815, and by *R. faecis* M72/1 (Fig. 1b). Xyloglucan could not be utilized for growth by any of the 10 strains tested, although xyloglucan overlay plates revealed that the *R. intestinalis* strains formed clear zones (Fig. S2).

Only *R. inulinivorans* L1-83 and *R. faecis* M72/1 were capable of utilizing 1,3–1,4-β-glucan whilst no strain utilized β-mannan (1,4-β-mannan backbone) or type 2 arabinogalactan (1,3-β-galactan backbone). Only *R. faecis* M72/1 was capable of utilizing type 1 arabinogalactan (1,4-β-galactan backbone) (Fig. 1c).

None of the strains was capable of using either type II or type III pig gastric mucin for growth (data not shown) and no degradation of type II or type III pig gastric mucin could be detected on overlay plates of the 10 strains (data not shown).

### Pan-genome of *Roseburia*/*E. rectale* group

Eleven genomes representing *E. rectale* and the four *Roseburia* spp. were investigated (Table 1). These comprised the 10 strains whose growth characteristics were compared in Fig. 1, with the addition of *E. rectale* ATCC33656 (Table S4). The genomes of *E. rectale* A1-86, ATCC33656 and M104/1, *R. intestinalis* L1-82, XB6B4 and M50/1, *R. inulinivorans* A2-194, and *R. hominis* A2-183 were sequenced previously and are publicly available in the GenBank database. In addition, high-quality draft genomes of *R. faecis* M72/1, *R. inulinivorans* L1-82 and *E. rectale* T1-815 were sequenced, assembled and automatically annotated in this work. The draft genomes were compared with the complete genome of *E. rectale* ATCC33656 (Fig. S3a). This revealed a high level of genome plasticity in the *Roseburia*/*E. rectale* group; in particular, sections of the *E. rectale* ATCC33656 genome were not present in the other genomes despite the fact that the genomes were of similar size (Table 1).

Core, variable and unique genes were identified by assigning all ORFs in the *Roseburia*/*E. rectale* pan-genome to orthologous groups (OGs). Orthologues present in all 11 strains were considered to be core genes, with all 11 orthologues forming a core OG; sequences with orthologues present in two to 10 strains were considered to be variable genes and sequences that were found only in one strain were considered unique genes. In this way, 794 core OGs, 5513 variable OGs and 7825 unique genes in the pan-genome (pan-genome details in Fig. S4) were identified. The distribution of genes between the core (mean 22.9 %, range 16.7–27.4 %), variable (mean 57.7 %, range 51.9–64.2 %) and unique (mean 19.4 %, range 10.9–29.4 %) genomes was similar in all strains.

### Detection of CAZymes

Predicted CAZyme-encoding genes in the pan-genome were identified to the protein family level *in silico* using HMMs representing conserved regions of all CAZyme families (Yin *et al.*, 2012). In addition, a protein database focusing on carbohydrate metabolism was established by collecting all the protein sequences in KEGG that had been assigned EC 3.2.1 (GH) and the *Roseburia*/*E. rectale* pan-genome was queried against this database using blastp. The combined results from these analyses resulted in the identification of 1840 CAZyme genes in the *Roseburia*/*E. rectale* pan-genome, including 932 GHs (Table S5), 503 glycosyltransferases, 243 carbohydrate esterases (CEs) and one polysaccharide lyase (Table S6). Only 74 (7.9 %) of these GHs were predicted to possess signal peptides (SPs) by SignalP software (Petersen *et al.*, 2011), indicating cell-bound or extracellular enzymes. These results are presented in Table S7. Alternative protein secretion of *Roseburia*/*E. rectale* pan-genome xylanases was also investigated using SecretomeP (Bendtsen *et al.*, 2005), but no new predicted secreted proteins were identified. Of the 932 GHs, 148 (16 %) were predicted to possess carbohydrate-binding modules (CBMs) (Table S8). GHs with CBMs were more likely to possess SPs (23 %) than GHs without CBMs (5 %).

The distribution of CAZymes, GHs and ‘all genes’ of the *Roseburia*/*E. rectale* pan-genome between the core, variable and unique genome fractions was compared (Fig. S5). There was a higher percentage of GHs and CAZymes (75 and 74 %, respectively) in the variable genome, compared with ‘all genes’ (58 %). Strikingly, only 26 out of a total of 538 OGs representing CAZymes within the pan-genome were found in all 11 genomes. These included 13 GH enzymes, including five GH13 and two putative oligosaccharide phosphorylases (Table S9).

The majority of GH OGs were therefore species-specific or strain-specific. For example, 107 CAZyme OGs (including 70 GHs) were found only in *R. intestinalis*, of which 31 (including 18 GHs) were present in all three *R. intestinalis* strains (Table S6). Meanwhile, 85 CAZyme OGs (including 24 GHs) were found only in *E. rectale*, of which only four CAZyme OGs (two GHs) were conserved in all four *E. rectale* strains. This represents considerable inter-strain variation within these species.

### Phylogenetic relationships within GH families

Within each genome, GH families were often represented by multiple genes, as exemplified by the 16 GH43 genes present in *R. intestinalis* L1-82 (Table S5). In order to investigate the sequence relationships more closely, protein sequence-based phylogenetic trees of GH13 (α-glucan degradation), GH32 (fructan degradation), GH10, GH43 and GH51 (plant cell wall polysaccharide degradation) were reconstructed. Many of the GHs clustered into strongly supported clades (bootstrap ≥ 90) that correlated largely with the annotations assigned to them by the KEGG GH database.

### GH13 family enzymes and starch utilization

**Fig. 2.**
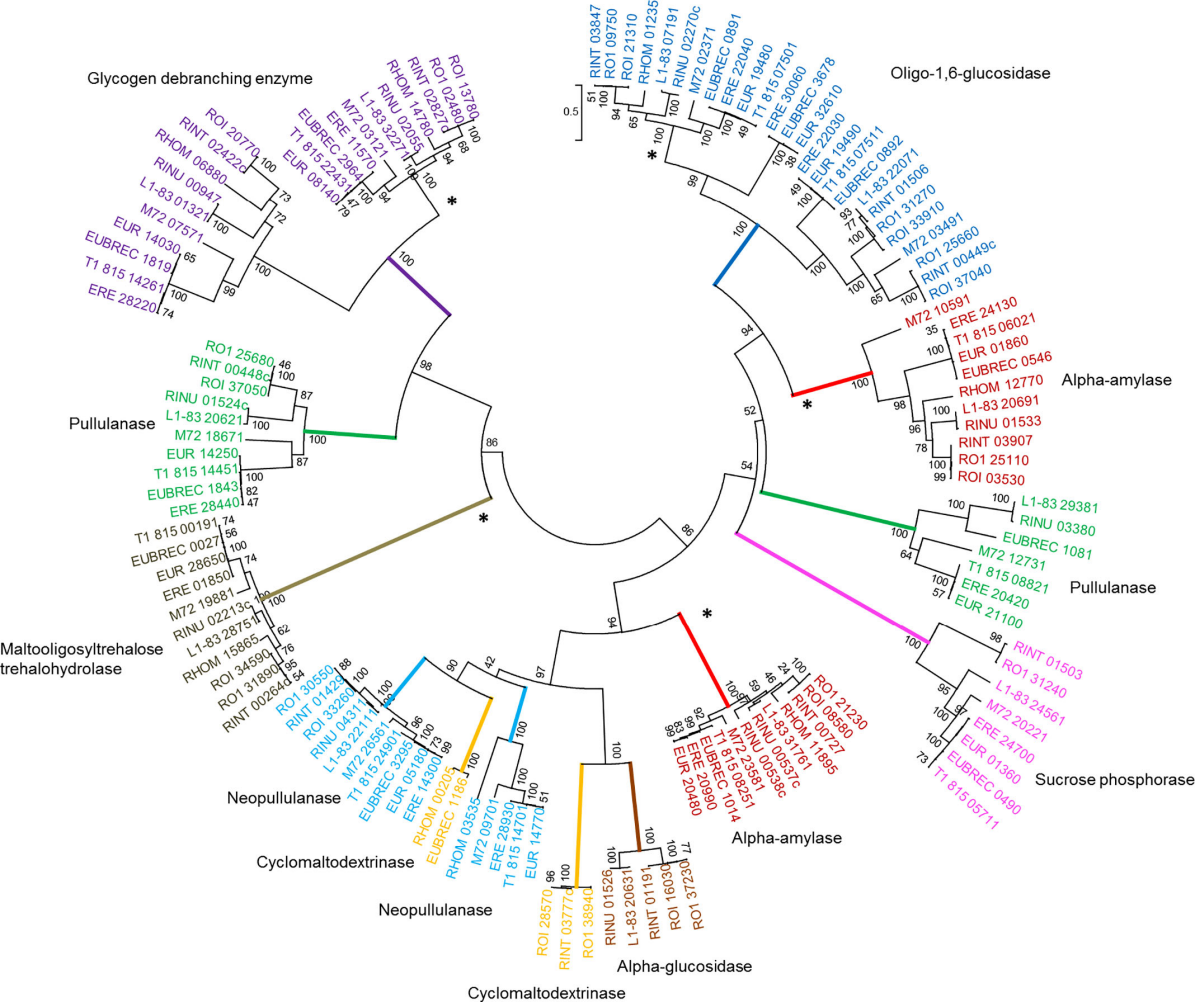
Phylogenetic tree of *Roseburia*/*E. rectale* GH13s. Gene names are colour-coded based on KEGG GH annotation. Strongly supported clades (bootstrap ≥ 90) are coloured at their most proximal branch. The branch colour corresponds to the KEGG GH annotation of the genes within the clade. Colour coding is as follows: neopullulanase (EC 3.2.1.135) (light blue), cyclomaltodextrinase (EC 3.2.1.54) (orange), α-glucosidase (EC 3.2.1.3) (brown), α-amylase (EC 3.2.1.1) (red), sucrose phosphorase (EC 3.2.1.7) (pink), oligo-1,6-glucosidase (EC 3.2.1.10) (blue), pullulanase (EC 3.2.1.41) (green), glycogen-debranching enzyme (EC 3.2.1.-) (purple) and malto-oligosyltrehalose trehalohydrolase (EC 3.2.1.141) (gold). Bootstrap values, expressed as a percentage of 1000 replications, are given at the branching nodes. This tree is unrooted and reconstructed using the maximum-likelihood method. The scale bar refers to the number of amino acid differences per position. Clades of core GH13s are indicated by asterisks at their most proximal branch.

The phylogenetic tree of the 130 GH13 sequences (Fig. 2) revealed clades similar to previously identified subfamilies that have tentatively assigned divergent functions (Stam *et al.*, 2006). A group of seven ‘pullulanases’ (the green clade at the right of tree, Fig. 2), which possess N-terminal SPs and (with the exception of M72_12731) putative C-terminal sortase signals, included the enzyme RINU_03380 which is responsible for the major amylase activity detected in *R. inulinivorans* A2-194 cell extracts (Ramsay *et al.*, 2006). The overexpressed gene product of RINU_03380 (Amy13C) hydrolysed α-1,4-glucan linkages in starch, but not α-1,6-glucan linkages (Ramsay *et al.*, 2006), whilst the related EUR_21100 enzyme from *E. rectale* has recently been shown to cleave α-1,4 linkages to release maltotetraose (Cockburn *et al.*, 2015), indicating that these enzymes are not true type 1 pullulanases. The enzymes in this clade also possess CBMs, either CBM26 (EUR_21100, ERE_20420, T1-815_08821 and M72_12731) or CBM41 (RINU_03380, L1-83_29381 and EUBREC_1081) (this work), which have both been described as starch-binding domains (Lammerts van Bueren *et al.*, 2004; Boraston *et al.*, 2006). Interestingly, whilst this clade was found in all four *E. rectale* strains, in both strains of *R. inulinivorans* and in *R. faecis*, no representative was present in *R. hominis* A2-183 or in the three *R. intestinalis* strains. *R. hominis* A2-183 also lacked representatives of two other GH13 OGs (annotated as a pullulanase and a neopullanase) that were found in the other 10 strains, which presumably explains why it was the only one of the 11 strains that was unable to grow with soluble starch as substrate. The major active extracellular GH13 enzyme (RINT_03777c) detected on a zymogram in *R. intestinalis* strains by Ramsay *et al.* (2006) belongs to a different clade than EUR_21100 and RINU_03380.

### GH32 family enzymes and utilization of fructans

Sequences belonging to GH32 (Fig. S6) were divided into five strongly supported clades: three β-fructofuranosidases, one levanase clade (unique to the *R. intestinalis* strains) and one divergent clade with no KEGG GH annotation (Fig. S6). The β-fructofuranosidase clade indicated by the red branch in Fig. S6 includes the *R. inulinivorans* A2-194 gene RINU_03877c, whose expression is upregulated 25-fold during growth on inulin, compared with growth on glucose, and encodes a β-fructofuranosidase that degrades intermediate- and long-chain fructan substrates (Scott *et al.*, 2011). This clade was found in all strains that were able to utilize inulin for growth and in only one other strain (*R. faecis* M72/1).

### GH families for utilization of hemicelluloses

Only five GH10 genes, which typically encode xylanases, were found in the *Roseburia*/*E. rectale* group pan-genome, two in *R. intestinalis* L1-82, and one each in *R. intestinalis* XB6B4, *E. rectale* T1-815 and *R. faecis* M72/1. Four of these GH10 genes (one in each strain) belonged to the same OG, and possessed two CBM9s and a SP. Hemicellulase activities are also found in the families GH43 (Fig. S7) and GH51 (Fig. S8). In total, 93 % (64 of 69) of the GH43s could be assigned into 11 clades, four of which were unique to *R. intestinalis* strains. The GH51 sequences fell into five putative α-l-arabinofuranosidase clades and one putative xylan-1,4-β-xylosidase clade (Fig. S8). When the GH43 and GH51 sequences were combined (Fig. S9), four GH43 family xylan-1,4-β-xylosidases clustered into the only xylan-1,4-β-xylosidase clade in GH51, suggesting some overlap between these families.

Only the three *R. intestinalis* strains encoded GH74 and GH26 enzymes, which are typically involved in utilization of xyloglucan and β-mannan, respectively (Table S5). The two *R. inulinivorans* strains lacked any representatives of GH10, GH26, GH43, GH51, consistent with their inability to utilize xylans or xylo-oligosaccharides for growth (Fig. 1) (Duncan *et al.*, 2006; Scott *et al.*, 2014).

### Determination of CUEs

**Fig. 3.**
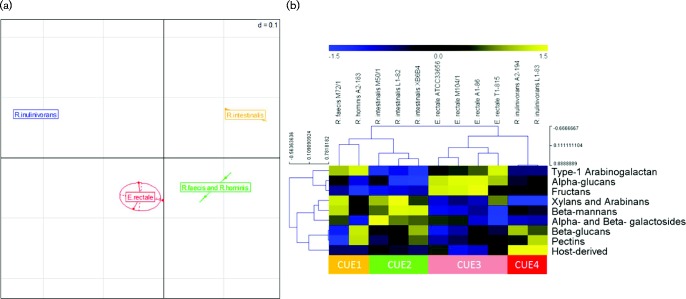
(a) Principal coordinate analysis of *Roseburia*/*E. rectale* strains based on complement of GH families and (b) heatmap showing CUEs. Values of a given GH family or carbohydrate set were taken as the number of these genes each genome possessed. In (a), coordinates were calculated using Kendal τ distance applied to the first five eigenvectors. *R. intestinalis* strains L1-82, M50/1 and XB6B4 (orange); *R. inulinivorans* strains A2-194 and L1-83 (blue); *R. faecis* M72/1 and *R. hominis* A2-183 (green); and *E. rectale* strains A1-86, T1-815, M104/1 and ATCC33656 (red) form separate clusters (*P* < 0.001, non-parametric multivariate ANOVA). In (b), CUEs were determined by complete linkage clustering using Kendall τ (as shown) and Spearman distance (not shown). GH53, an endo-1,4-β-galactanase that cleaves the β-1,4-d-galactosidic linkages in type I arabinogalactans, was assigned to the carbohydrate set ‘Type 1 arabinogalactan’ and is excluded from ‘Xylans and Arabinans’, ‘Pectins’ and ‘Alpha- and Beta-galactosides’. CUE1 consists of *R. faecis* M72/1 and *R. hominis* A2-183. CUE2 consists of *R. intestinalis* strains L1-82, M50/1 and XB6B4. CUE3 consists of *E. rectale* strains A1-86, T1-815, M104/1 and ATCC33656. CUE4 consists of *R. inulinivorans* strains A2-194 and L1-83. GH assignment to each carbohydrate set is described in Table S6.

Principal coordinate analysis revealed that the 11 strains formed four statistically significant (*P* < 0.001) clusters based on their GH family complement (Fig. 3a). Three of these clusters were specific to *E. rectale*, *R. intestinalis* and *R. inulinivorans*. The single strains of *R. faecis* M72/1 and *R. hominis* A2-183 did not fall into these three clusters. The GHs of each strain were also assigned into sets, based on their predicted activity against carbohydrate substrates (Table S2). Strain clustering was retained after these data transformations (Fig. S10) and resulted in four statistically significant carbohydrate set-based clusters that we call carbohydrate utilization ecotypes (CUEs; Fig. 3b). CUE1, which includes *R. hominis* A2-183 and *R. faecis* M72/1, was enriched in type 1 arabinogalactan-degrading genes (*P* = 0.03).

CUE2, which includes the three *R. intestinalis* strains, was enriched for xylan, arabinan, pectin, β-mannan and galactose sugar degradation genes (*P* < 0.03). CUE3, which includes all four *E. rectale* strains, was enriched for fructan degradation genes (*P* = 0.02) and CUE4, which includes both *R. inulinivorans* strains, was enriched for host-derived carbohydrate degradation genes, such as mucin glycans (*P* = 0.04). The relationship between predicted CUE and actual growth behaviour will be discussed below.

### PULs in the *Roseburia*/*E. rectale* group

PULs are an important feature of *Bacteroides* genomes (Martens *et al.*, 2008; Larsbrink *et al.*, 2014; Cuskin *et al.*, 2015) and it was therefore decided to investigate the *Roseburia*/*E. rectale* group genomes for the presence of gene clusters dedicated to carbohydrate utilization.

*R. intestinalis* XB6B4 was selected for detailed analysis because it possessed the second highest number of GHs (131 GHs compared with 146 in *R. intestinalis* L1-82), but the draft genome had a smaller number of contigs. PULs of *Bacteroidetes* are defined as possessing, at minimum, a TonB-dependent transporter/SusD family lipoprotein-encoding gene pair. As Gram-positive bacteria lack outer membrane transporters, a new definition for PULs is required for these organisms. Here, we define a Gram-positive PUL (gpPUL) as being a locus encoding, at minimum, one polysaccharide-degrading enzyme, a carbohydrate transport system and a transcriptional regulator.

*R. intestinalis* XB6B4, which was originally selectively isolated for its high xylan-degrading activity (Chassard *et al.*, 2007), was predicted to possess 33 gpPULs. Predicted carbohydrate transport systems were adjacent to GH genes in gpPULs. Of the 35 carbohydrate transporters identified in *R. intestinalis* XB6B4 gpPULs, 26 (79 %) were ATP-binding cassette (ABC) transporters, 7 (20 %) were glycoside–pentoside–hexuronide (GPH) : cation symporter family transporters, one was a major facilitator superfamily (MFS) transporter and one was a phosphotransferase system (PTS) transporter. No evidence of SusC and SusD homologues, which are the carbohydrate transporters almost universally observed in PULs from the *Bacteroidetes*, could be found in *R. intestinalis* XB6B4.

Many of the gpPULs in *R. intestinalis* XB6B4 encoded GH and CE enzymes that can target multiple carbohydrates, making functional predictions difficult. Despite this, the likely target substrate(s) of 10 (out of 33) gpPULs could be predicted with reasonable confidence based on the GHs and CEs present (Table 2): these included a gpPUL concerned with utilization of pectin and xylan (Ros-2), arabinoxylan (Ros-6 and Ros-7), arabinan (Ros-8), arabinogalactan (Ros-5), glycogen (Ros-4), and glucomannan/galactomannan (Ros-3). *R. intestinalis* XB6B4 also had gpPULs predicted to utilize *O*-linked mucus glycans (Ros-1) and *N*-linked mucus glycans (Ros-10). The remaining gpPUL in *R. intestinalis* XB6B4 (Ros-9) was predicted to encode genes for the utilization of arabinogalactan and glucomannan.

**Table 2.** gpPULs identified in *R. intestinalis* XB6B4 and *E. rectale* A1-86 for which the substrate target(s) could be confidently predicted The carbohydrates utilized by these gpPULs were predicted by their complement of GHs and CEs. ABC transporters, GPH : cation symporter family transporters and MFS transporters were predicted to mediate carbohydrate transport for some of the gpPULs. Transcriptional regulators were identified similar to those of the l-arabinose operon (AraC), lactose operon (LacI), arsenic resistance operon (AsrR), methyl-accepting chemotaxis sensory transducer (MCST), tetracycline resistance genes (TetR) and *N*-acetyl-d-galactosamine operon (NagC), histidine kinase (HK), response regulator with AraC-like DNA-binding domain and CheY-like receiver domain (RR[AraC-CheY]) and response regulator with LytTR-like DNA-binding domain (RR[LytTR]). The gpPULs of *R. intestinalis* XB6B4 and *E. rectale* A1-86 possess the prefixes ‘Ros-’ and ‘Eub-’, respectively.

**PUL**	**Predicted substrate**	**GHs and CEs**	**Transport system**	**Transcriptional regulation**
Ros-1	*O*-linked mucus glycans	GH29, GH42	ABC	HK
Ros-2	Pectin and xylan	GH28, 2 GH43, CE12	ABC	AraC
Ros-3	Gluco- and galactomannan	GH1, GH36, GH76, GH113, 2 GH130, CE2, CE3	ABC	AraC, LacI
Ros-4	Glycogen	GH13, GH77, GH78/15,	ABC	AsrR, LacI
Ros-5	Xylan and arabinogalactan	GH2, GH3, GH8, GH42, GH43, GH53, GH115,	2 × ABC	HK, RR[AraC–CheY], LacI, AraC
Ros-6	Arabinoxylan	2 GH39, GH43/51, GH43, 2 GH51, GH120, 2 CE1	ABC	LacI, AraC
Ros-7	Arabinoxylan	GH25, 2 GH43, GH51	ABC and GPH	AraC, MCST, RR[LytTR], HK
Ros-8	Arabinan	GH51, GH127	ABC	TetR
Ros-9	Arabinogalactan and glucomannan	GH2, GH5, GH53, GH130, CE1F	2 × ABC	LacI
Ros-10	*N*-linked mucus glycans	GH3, GH38, GH85, GH125, GH130, GH20, CE1,	ABC and MFS	2 LacI, 2 NagC
Eub-1	Starch	2 GH31	ABC	LacI
Eub-2	Arabinogalactan	GH2, GH53	GPH	AraC
Eub-3	Fructans	GH32	ABC	LacI
Eub-4	Fructans	GH32	ABC	LacI

*E. rectale* A1-86 was also selected for detailed analysis because *E. rectale* is the most abundant species of the *Roseburia*/*E. rectale* group present in the colonic microbiota (Walker *et al.*, 2011; Louis *et al.*, 2010). Again, SusC and SusD homologues were not identified in the genome of *E. rectale* A1-86, but alternative carbohydrate transport systems were adjacent to GH genes in gpPULs. This strain was predicted to possess 15 gpPULs. Of the 18 carbohydrate transporters identified in *E. rectale* A1-86, 10 (56 %) were ABC transporters, six (33 %) were GPH : cation symporter family transporters and two (11 %) were PTS transporters. The likely carbohydrate targets of the four of these gpPULs that could be predicted with reasonable confidence were starch (Eub-1) and fructan (Eub-3 and Eub-4) (Table 2). *E. rectale* A1-86 also possessed a gpPUL predicted to utilize arabinogalactan (Eub-2). This gpPUL was orthologous to Ros-5, possessed by *R. intestinalis* XB6B4.

**Fig. 4.**
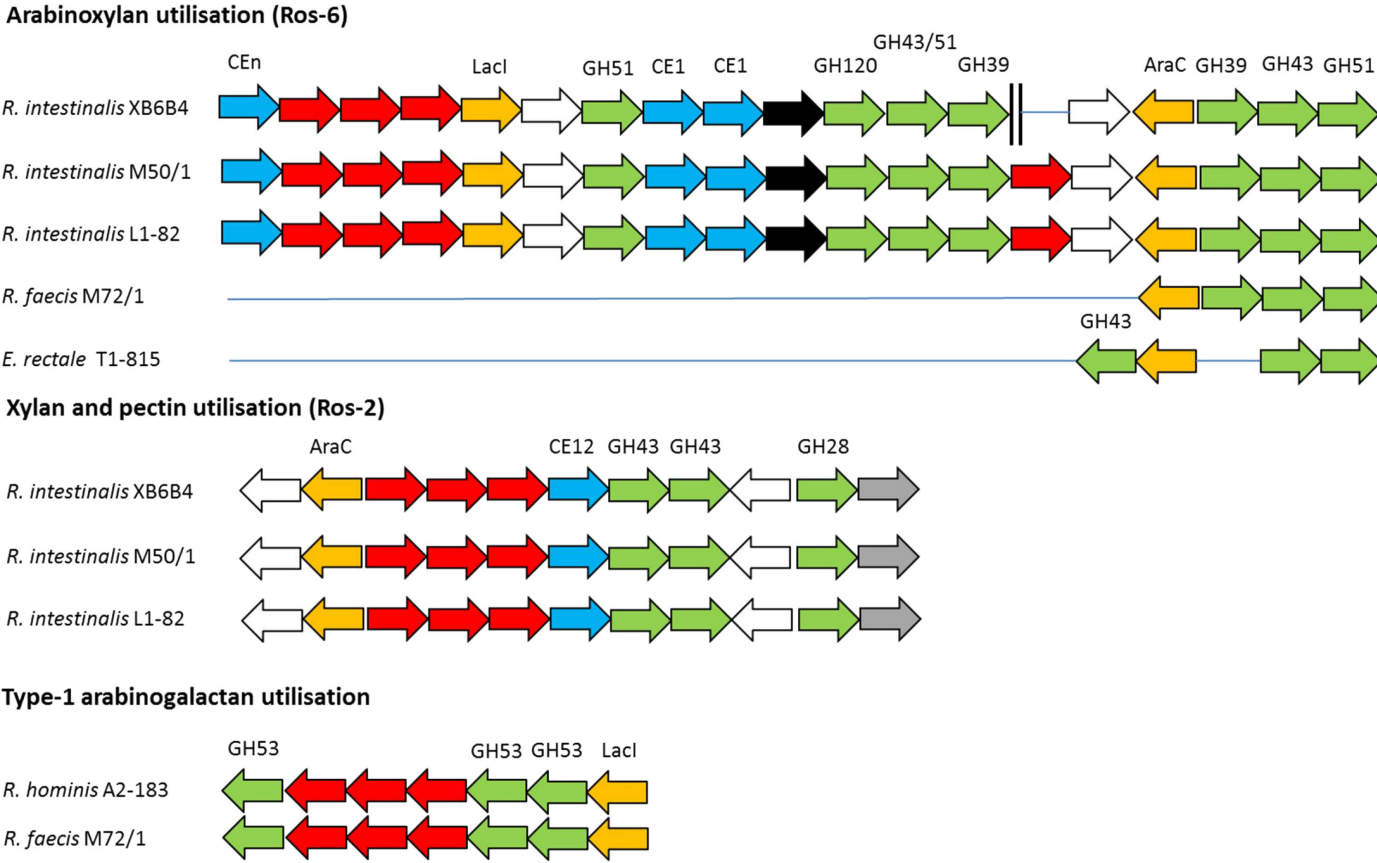
Schematic representation of gpPULs concerned with xylan and arabinogalactan utilization. Glycoside hydrolase genes are coloured green. Carbohydrate esterase genes are coloured blue. ABC-transporter system component genes are coloured red. Transcriptional regulator genes are coloured yellow. Uncharacterized transporter genes are coloured black. Hypothetical genes are coloured white. Xylose isomerase genes are coloured grey. Two parallel black bars between genes indicate sections that are separated in the genome sequence. *Roseburia*/*E. rectale* strains not represented in the diagram lack an orthologous gpPUL. Genes located vertically to each other are orthologues. Solid blue lines between genes are for easy visual comparison of the genes between species and do not represent real gaps in the genome. Locus tags of gpPULs are listed in Table S7.

Specific gpPULs of interest were selected for comparison across strains. The predicted xylan utilization gpPUL Ros-6 showed well-conserved gene order for the three *R. intestinalis* strains, but *R. faecis* M72/1 and *E. rectale* T1-815 contained only a few of the genes from this gpPUL (Fig. 4a), and this gpPUL was completely absent in the other *E. rectale* strains, *R. inulinivorans* and *R. hominis*.

Ros-2 was unique to *R. intestinalis* and its gene order was perfectly conserved between the three *R. intestinalis* strains. This gpPUL possessed two GH43 genes predicted to encode xylan-1,4-β-xylosidases (EC 3.2.1.37), a xylose isomerase gene, a CE12 gene (xylan/pectin esterases) and a GH28 gene predicted to encode a polygalacturonase (EC 3.2.1.15). Ros-2 also encoded an AraC-like transcriptional regulator and an ABC transporter system (Fig. 4a). *R. hominis* A2-183 and *R. faecis* M72/1 both possessed a predicted arabinogalactan gpPUL that was absent in the other nine strains of the *Roseburia*/*E. rectale* pan-genome (Fig. 4b, Table S10). This gpPUL consisted of three GH53 genes predicted to encode arabinogalactan endo-1,4-β-galactosidases (EC 3.2.1.89), two of which possessed a CBM61 (1,4-β-galactan binding; Cid *et al.*, 2010) and a SP.

**Fig. 5.**
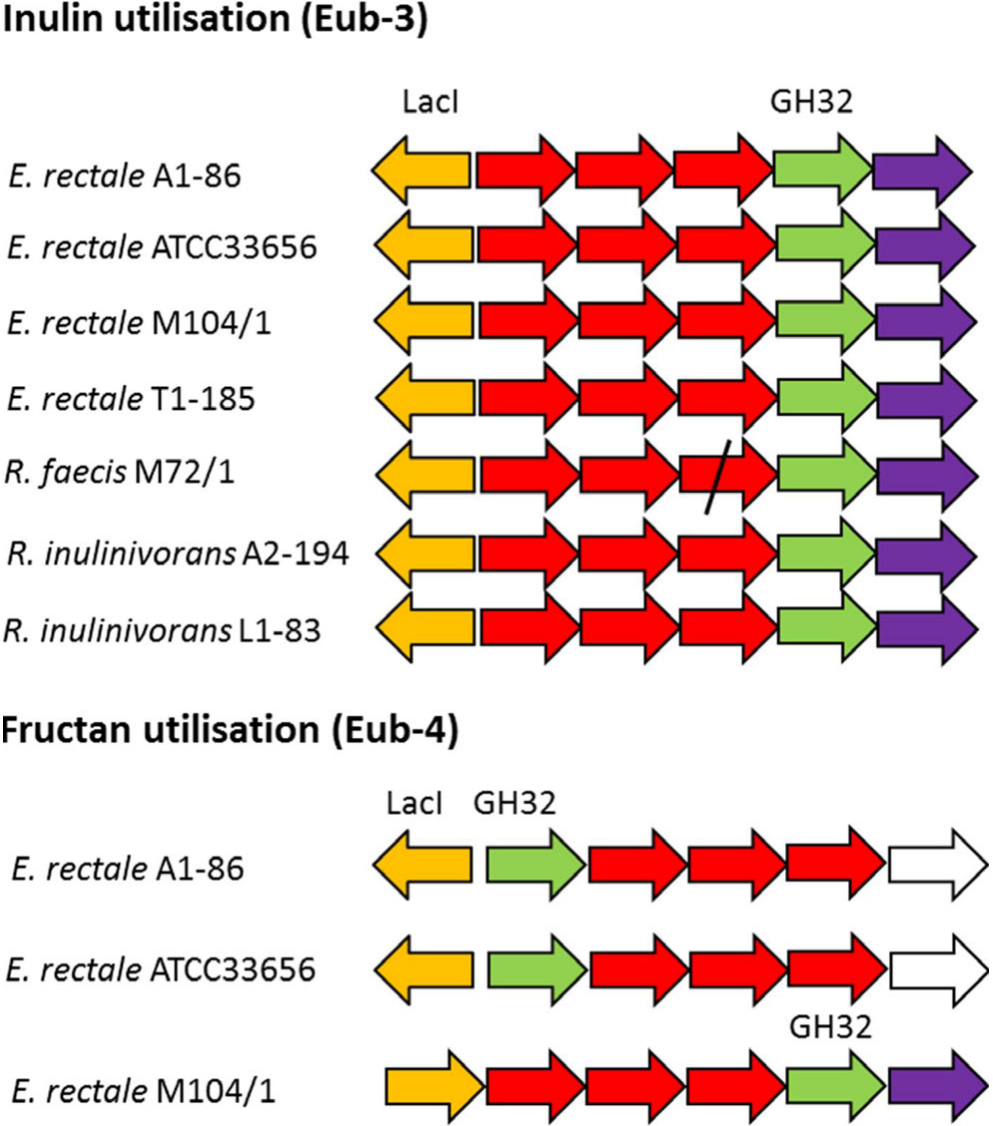
Schematic representation of gpPULs concerned with fructan utilization. GH genes are coloured green. ABC transporter system component genes are coloured red. Transcriptional regulator genes are coloured yellow. Fructokinase genes are coloured purple. Hypothetical genes are coloured white. Any of the 11 *Roseburia*/*E. rectale* strains not represented in the diagram lack an orthologous gpPUL. Genes located vertically to each other are orthologues. The diagonal line through the *R. faecis* gene represents a frameshift mutation. Locus tags of gpPULs are listed in Table S7.

The predicted inulin utilization gpPUL Eub-3 was present in all *E. rectale* and *R. inulinivorans* strains, and in *R. faecis* M72/1, whilst *E. rectale* strains A1-86, ATCC33656 and M104/1 also possessed a second fructan gpPUL Eub-4 (Fig. 5, Table S10). Of the 10 strains tested for growth on inulin, all strains possessing Eub-3 were capable of utilizing inulin for growth, with the exception of *R. faecis* M72/1 (Fig. 1a). The *R. faecis* M72/1 Eub-3 contained a substitution SNP (C replaced with T) at nucleotide 381 of an ABC transporter permease, predicted to result in a truncated protein and likely explaining the inability of *R. faecis* M72/1 to grow on inulin. This mutation, first observed in the genome sequence, was subsequently confirmed by targeted Sanger sequencing. None of the *R. intestinalis* of *R. hominis* strains, which lack Eub-3, were capable of utilizing inulin.

Eub-4 possesses a GH32 gene predicted to encode a β-fructofuranosidase (EC 3.2.1.26). Although the GH32 genes in this gpPUL were predicted to be orthologues, the *E. rectale* M104/1 gene lacked the CBM66 (binding of terminal fructose moiety of levantriose; Cuskin *et al.*, 2012) present in the GH32 genes of *E. rectale* A1-86 and ATCC33656.

**Fig. 6.**
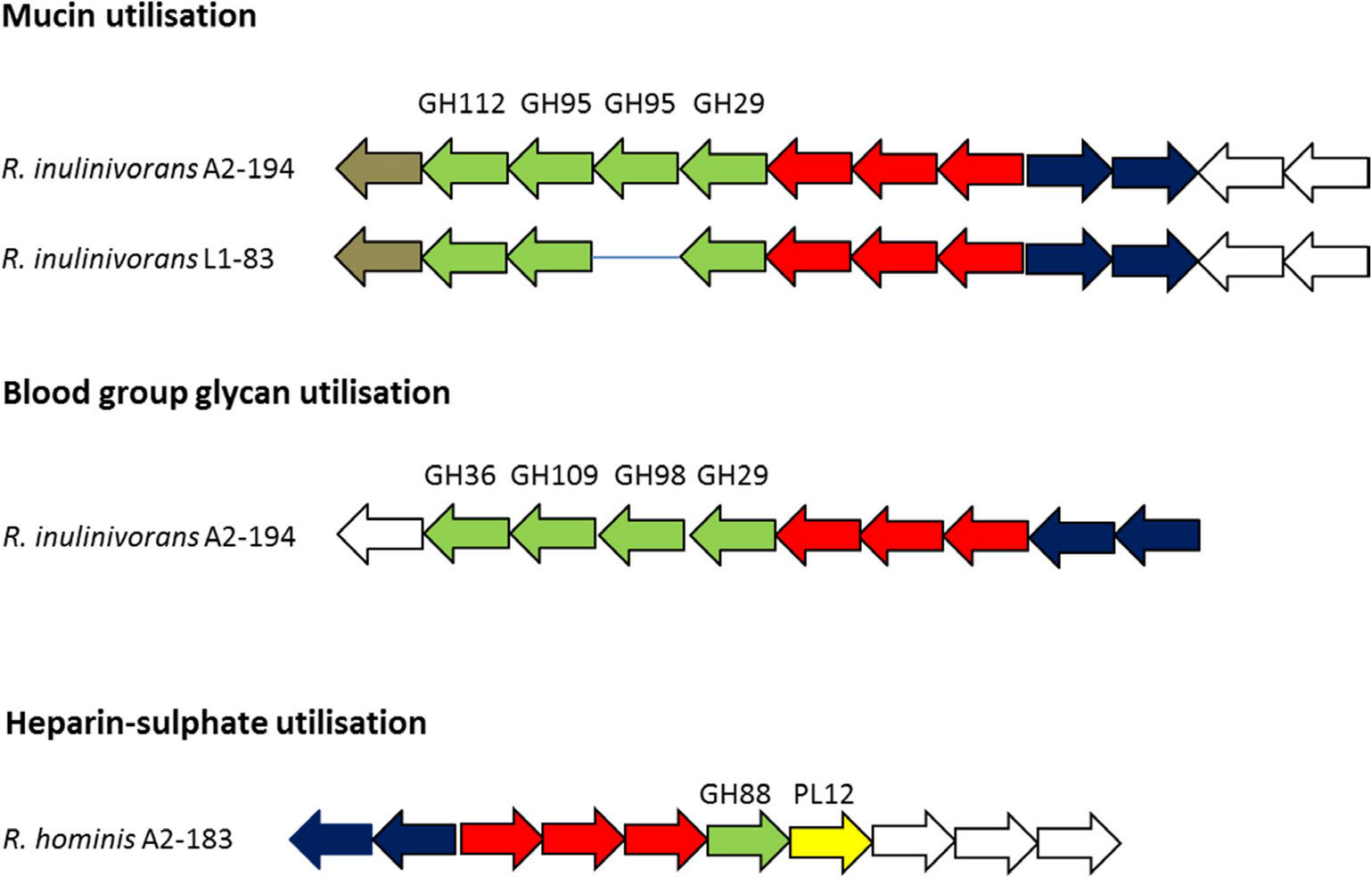
Schematic representation of gpPULs concerned with host-derived carbohydrates. GH genes are coloured green. ABC transporter system component genes are coloured red. The polysaccharide lyase gene is coloured bright yellow. Mucin desulphatase genes are coloured gold. Hypothetical genes are coloured white. Two-component signal transduction component genes consisting of a histidine kinase and a response regulator containing a CheY-like receiver domain and an AraC-like DNA-binding domain are coloured navy. Solid blue lines between genes are for easy visual comparison of the genes between species and do not represent real gaps in the genome. Any of the 11 *Roseburia*/*E. rectale* strains not represented in the diagram lack an orthologous gpPUL. Locus tags of gpPULs are listed in Table S7.

The two *R. inulinivorans* strains possessed a predicted mucin gpPUL that is absent in the other nine strains (Fig. 6, Table S10), which encoded a mucin desulphatase, four mucin-degrading GHs and an ABC transporter system. *R. inulinivorans* A2-194 also possessed a predicted blood group glycan gpPUL that was absent in the other strains (Fig. 6). This gpPUL contained four GH genes predicted to encode enzymes for the degradation of blood group glycans, including a SP possessing blood-group endo-1,4-β-galactosidase (EC 3.2.1.102) harbouring two CBM51 domains – a CBM family shown to bind blood group A/B antigens in *Clostridium perfringens* (Gregg *et al.*, 2008). This gpPUL was also predicted to encode a GH109 enzyme, but particular caution should be taken when annotating members of this GH family *in silico*, as the sequences of true GH109 enzymes are highly similar to oxidoreductases that do not degrade carbohydrates. An additional gpPUL, dedicated to the utilization of fucose as a growth substrate, was previously identified and shown to be inducible in *R. inulinivorans* A2-194 during growth on fucose (Scott *et al.*, 2006).

The only polysaccharide lyase found in the *Roseburia*/*E. rectale* pan-genome was encoded by *R. hominis* A2-183. This gene was part of a gpPUL predicted to utilize heparin sulphate (components of extracellular matrix and cell surface proteoglycans) (Fig. 6).

## Discussion

Whilst carbohydrate utilization in the Gram-negative *Bacteroidetes* phylum has been investigated extensively and is well understood (D'Elia & Salyers, 1996; Reeves *et al.*, 1997; Shipman *et al.*, 2000; Martens *et al.*, 2008, 2011; McNulty *et al.*, 2013; Larsbrink *et al.*, 2014), the present work represents the first detailed analysis of carbohydrate utilization genes and their organization within a dominant group of human colonic *Firmicutes*. The 11 strains of *Roseburia* and *E. rectale* (the ‘*Roseburia*/*E. rectale* group’) examined here encoded a mean number of 85 GHs per genome. This is much higher than the mean number of GHs reported per genome for *Firmicutes* (40 GHs), but much lower than the mean number of GHs per genome for *Bacteroidetes* (130 GHs) in a ‘mini-microbiome’ of human colonic bacteria (El Kaoutari *et al.*, 2013). The possession of relatively large numbers of GH genes is in general agreement with findings from human dietary studies that illustrate the dependence of *Roseburia* and *E. rectale* populations upon dietary sources of carbohydrate (Duncan *et al.*, 2007; Martínez *et al.*, 2010; Walker *et al.*, 2011; Salonen *et al.*, 2014).

A fundamental feature of carbohydrate utilization genes in *Bacteroides* spp. is their clustering into genomic regions, termed PULs. Polysaccharide utilization in *Bacteroidetes* involves limited extracellular cleavage of polysaccharides, followed by the binding and translocation into the periplasm of the released oligosaccharides via outer membrane Sus protein homologues. In addition to GH genes, these PULs encode the Sus proteins and also transcriptional regulation systems (most frequently hybrid two-component regulators) that respond to the presence of specific carbohydrates (Martens *et al.*, 2011).

We report here that PULs are an equally important feature of genome organization in the *Roseburia*/*E. rectale* group of *Firmicutes*. The genome of *R. intestinalis* XB6B4 was found to contain 33 gpPULs, which contained 106 of its 131 GH genes. As in *Bacteroides*, these gpPULs appear to be substrate-specific, and include linked transport systems and regulatory genes. ABC transport systems predominate, accounting for 79 % of transporters within gpPULs in *R. intestinalis* XB6B4 and 56 % in *E. rectale* A1-86, with cation symporters and PTS systems found in smaller numbers. No evidence was found for close homologues of the *Bacteroidetes* Sus proteins; binding of polysaccharides in the *Roseburia* group therefore seems likely to involve the CBMs present in many GH enzymes, whilst ABC transport components are assumed to mediate binding of oligosaccharides prior to transport in the majority of cases. gpPUL-encoded GHs in *E. rectale* and *R. inulinivorans* are known to be highly inducible (Scott *et al.*, 2011; Cockburn *et al.*, 2015), as is seen in *Bacteroides* (Martens *et al.*, 2008, 2011; McNulty *et al.*, 2013). The adjacent transcriptional regulators of the *Roseburia*/*E. rectale* group tend to be LacI- and AraC-type proteins with only a few examples of the hybrid two-component system transcriptional regulators. Hybrid two-component system transcriptional regulators and extracytoplasmic function sigma factors are, however, the most frequently observed regulators in *Bacteroides* PULs (Sonnenburg *et al.*, 2006). The differences revealed here in membrane organization, SPs, transport and regulatory systems all suggest that the detailed organization and regulation of degradative enzymes differs in this group of Gram-positive bacteria from that in *Bacteroides* spp. It is also apparent that these features may differ substantially in a second family of *Firmicutes* that is highly abundant in the human colon, i.e. the *Ruminococcaceae* (Wegmann *et al.*, 2014; Ben David *et al.*, 2015; Ze *et al.*, 2015).

Another important conclusion of the present study is that different species of the *Roseburia*/*E. rectale* group show considerable specialization in their abilities to utilize different carbohydrate substrates. Based initially on the CAZyme content of their genomes, these strains could be assigned to CUEs that consisted, in three out of four cases, entirely of members of a single species. The remaining CUE (CUE1) consists of the single available genome sequences for *R. hominis* and *R. faecis*. Our data suggest that most members of the *Roseburia*/*E. rectale* group share a core capacity to utilize starch and fructo-oligosaccharides, with only *R. hominis* A2-183 less capable of utilizing both. In addition, however, *R. intestinalis* is predicted to specialize in the degradation of plant cell wall matrix polysaccharides (e.g. arabinoxylan), *R. inulinivorans* in degrading host-derived carbohydrates, and *R. hominis* and *R. faecis* in type 1 arabinogalactan degradation. The correspondence between the genome-predicted ecotype and the observed growth of strains on different substrates was not always straightforward and requires some comment. The enrichment of genes associated with xylan breakdown in *R. intestinalis* strains corresponded well with their ability to grow on arabinoxylan and xylo-oligosaccharides. However, two *E. rectale* strains lacking many of these gpPULs were also able to grow on arabinoxylan and xylo-oligosaccharides. This might perhaps be explained by an as yet undiscovered xylanase or the utilization of different breakdown products, e.g. removal of arabinose substituents as opposed to cleavage of the main xylan chain. In addition, production of a GH74 enzyme by *R. intestinalis* strains and enzymic activity against xyloglucan did not correlate with growth on this substrate, presumably because hydrolysis products were not utilized. Furthermore, possession of hydrolases concerned with particular host glycans did not lead to growth on mucin in any of the strains, presumably because this requires a wider repertoire of enzymic specificities. Particularly in the case of mucin and plant structural polysaccharides, it should be recognized that the complexity and variability of the substrates make simple predictions from genomic data tentative. Nevertheless, *in vivo* evidence from human studies confirms that these species show variation with respect to dietary carbohydrate supplementation and individual microbiota composition (Louis *et al.*, 2010; Martínez *et al.*, 2010; Walker *et al*., 2011; Salonen *et al.*, 2014). Work by Louis *et al.* (2010) based on amplification of the butyryl-CoA : acetate CoA transferase gene revealed striking inter-individual variation within the *Roseburia*/*E. rectale* group, with *E. rectale* dominant in six individuals, *R. faecis* in two individuals and *R. inulinivorans* in one individual. The nutritional specialization revealed by the present work, assuming variations in dietary intakes, provides a plausible explanation for such differences.

The percentage of *Roseburia*/*E. rectale* GHs possessing SPs was unusually low at only 7.9 %. This is in marked contrast with some other human colonic bacteria, such as *Bacteroidetes*, that are predicted to secrete ∼85 % of their GHs (El Kaoutari *et al.*, 2013). El Kaoutari *et al.* (2013) also reported that only 19 % of *Firmicutes* GHs in their ‘mini-microbiome’ possessed SPs, although SPs are found in a high proportion of GHs in *Ruminococcus* spp. from the rumen and human colon (Rincon *et al.*, 2010; Wegmann *et al.*, 2014). The low percentage of SPs among GH enzymes might therefore be a feature mainly of the *Lachnospiraceae* – the most abundant family of *Firmicutes* in the human colon. It remains to be established whether GHs in the *Roseburia/E. rectale* group of *Lachnospiraceae* that lack SPs are mostly intracellular, or whether (as seems more likely) many possess alternative signal sequences enabling secretion or positioning within the cell membrane. Of the two amylases found to be upregulated by growth on starch in *E. rectale*, one possessed a SP and the other a hydrophobic region suggesting a possible membrane location (Cockburn *et al.*, 2015). Previous analysis of amylopullulanases in *R. inulinivorans* identified an inducible multidomain enzyme involved in starch degradation that had a SP and a hydrophobic region, as well as both catalytic and carbohydrate-binding domains (Ramsay *et al.*, 2006; Scott *et al.*, 2011). Our work revealed the SP-possessing amylases of *R. inulinivorans* and *E. rectale* to be orthologues of each other, with *R. faecis* M72/1 and all strains of both *R. inulinivorans* and *E. rectale* possessing a copy of this gene.

In conclusion, understanding the impact of diet on the human gut microbiota and gut metabolism requires a far better understanding of these important but little-studied groups of *Firmicutes* bacteria that appear to make a highly significant contribution to the fermentation of polysaccharides. This work has shown that this can come initially from comparative genome analysis that can subsequently be used to guide functional studies (Flint *et al.*, 2008).

## Supplementary Data

1353 Supplementary Data
