# Supplementary material for: Polysaccharide utilization loci and nutritional specialization in a dominant group of butyrate-producing human colonic Firmicutes
Source: Microb Genom. 2016 Feb 9;2(2):e000043. doi: 10.1099/mgen.0.000043 (PMC5320581; doi:10.1099/mgen.0.000043)
Supplement: Supplementary file 1 — Supplementary Data [file mgen-02-43-s001.pdf]

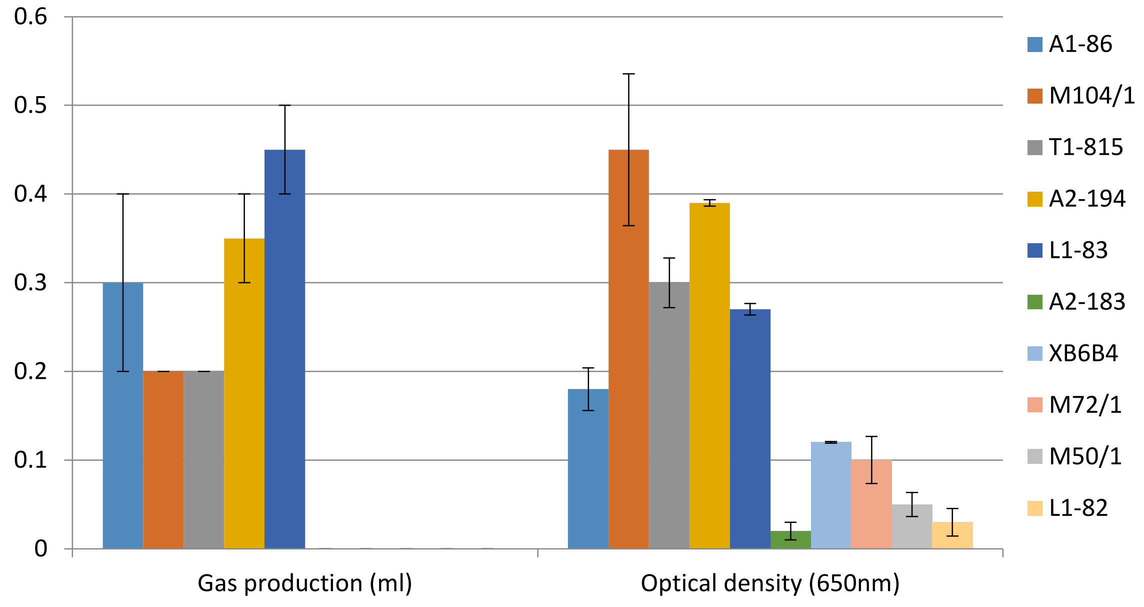

Figure S1. Growth of *Roseburia*/*E. rectale* strains on 0.5% inulin in Hungate tubes. Data plotted are the average maximum OD_{650} readings of three replicates ± standard deviation. Gas production was measured as milliliters of displacement in a 1 ml syringe.

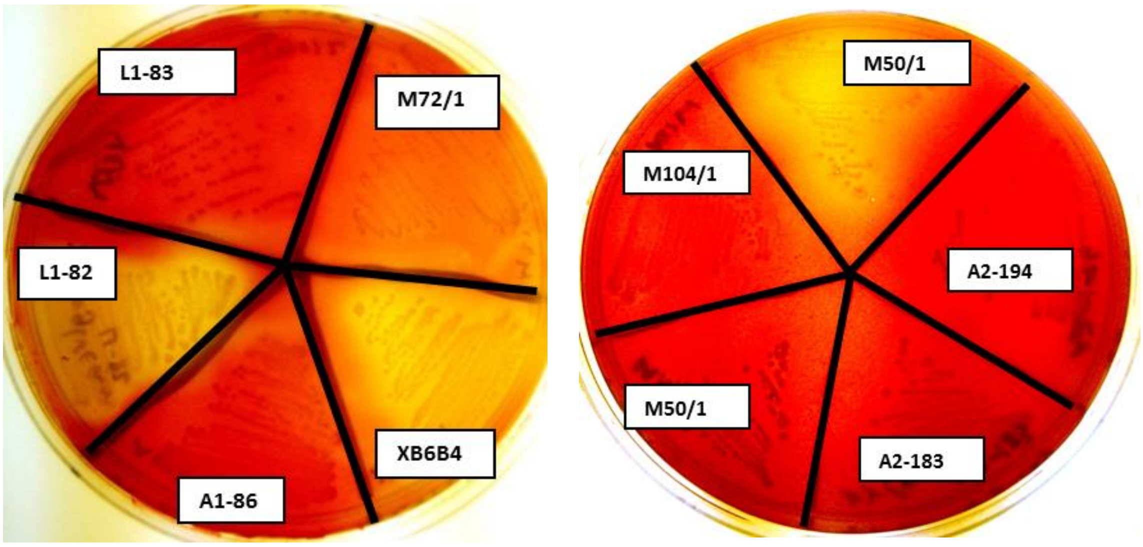

Figure S2. Xyloglucan degradative activity of *Roseburia*/*E. rectale* strains. Agarose solutions containing xyloglucan were overlaid onto agar plates containing colonies of the *Roseburia*/*E. rectale* strains. These plates were incubated anaerobically for 24 h and degradation of xyloglucan was assessed by congo red staining.

a. b.

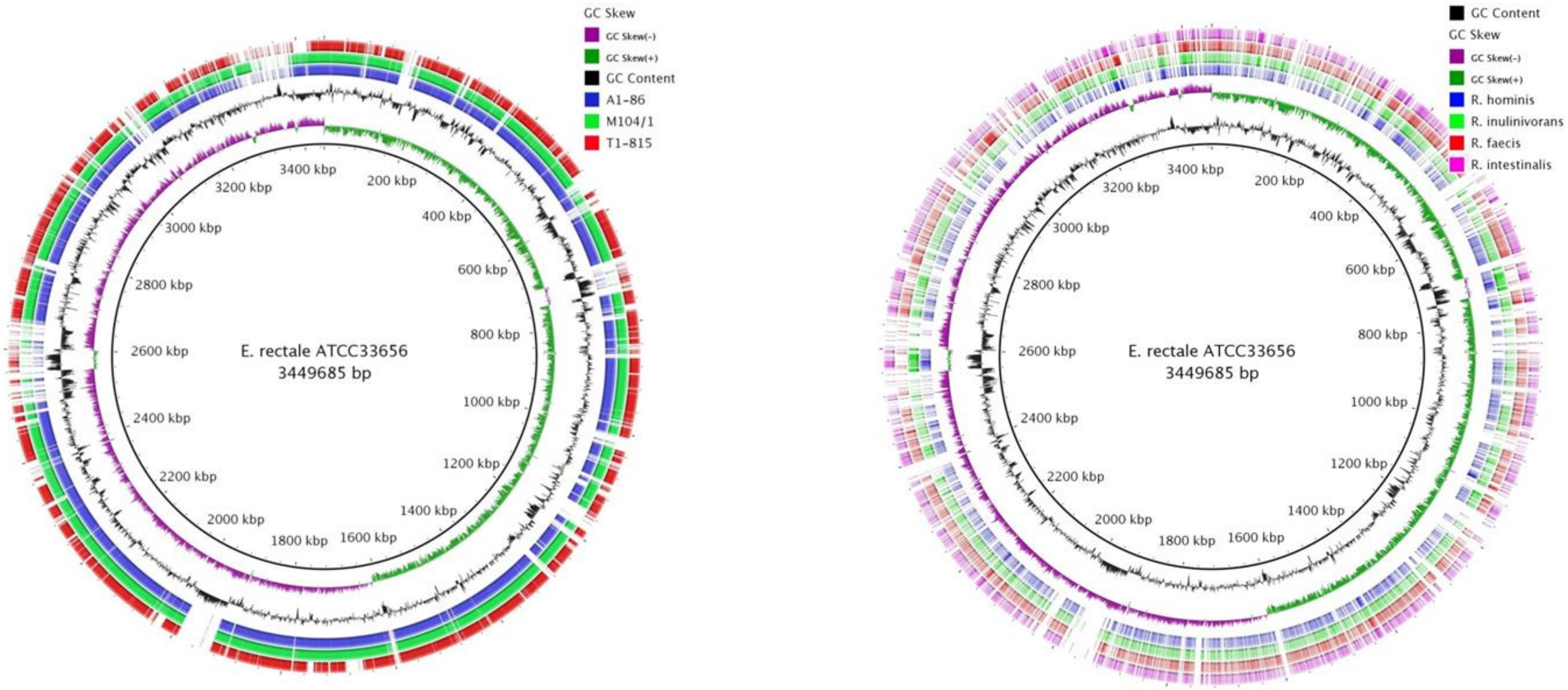

Figure S3. Intraspecies (a) and interspecies (b) whole-genome comparison of the *Roseburia*/*E. rectale* group. a) The complete genome sequence of *E. rectale* ATCC33656 was compared by BLASTn against the draft genome sequences of *E. rectale* A1-86, M104/1 and T1-815 using BLAST Ring Image Generator (BRIG). **b)** The complete genome sequence of *E. rectale* ATCC33656 was compared by BLASTn against the genome sequences of *R. hominis* A2-183 (R. hominis), *R. inulinivorans* A2-194 (R. inulinivorans), *R. faecis* M72/1 (R. faecis) and *R. intestinalis* L1-82 (R. intestinalis) using BLAST Ring Image Generator (BRIG). Genome accession numbers are indicated in Table S4. When the protein-encoding genes in the large strain-specific section between 2559-2602 kbp in *E. rectale* ATCC33656 were compared to the *Roseburia/E. rectale* pan-genome, the majority lacked orthologs in any of the other ten strains.

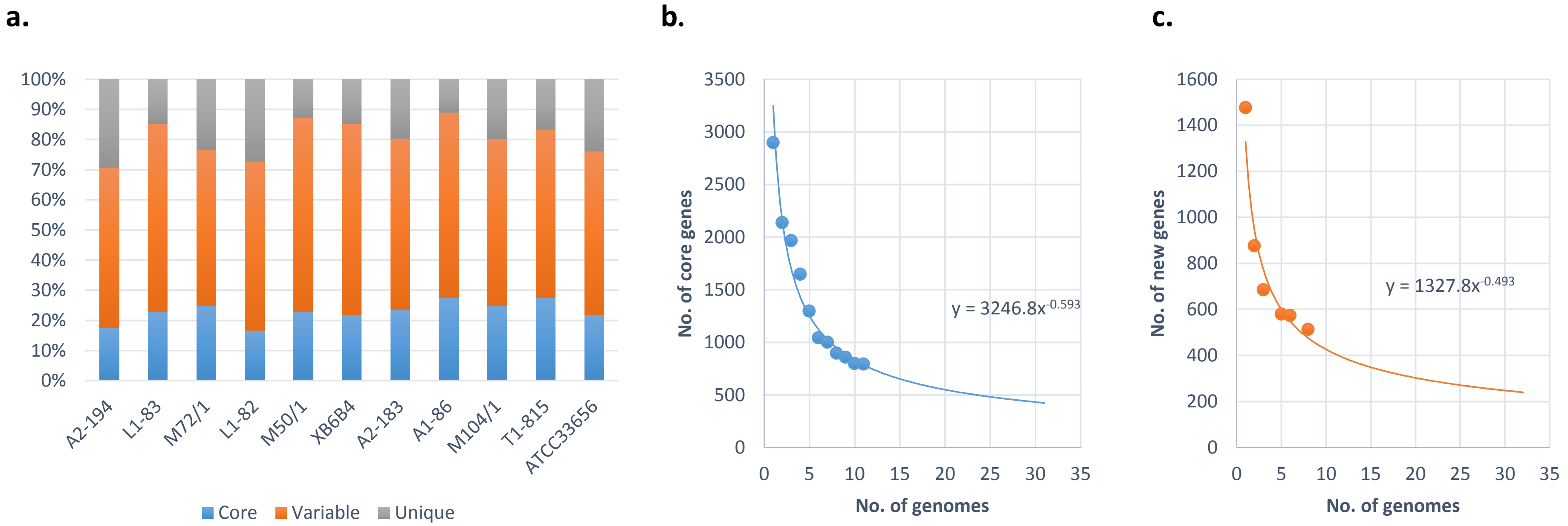

Figure S4. Pan-genome details. a. The percentage of core genes (genes conserved in all 11 strains), variable genes (genes conserved in 2-10 strains) and unique genes (genes present in only a single strain) in the genomes of *R. inulinivorans* (A2-194 and L1-83), *R. faecis* (M72/1), *R. intestinalis* (L1-82, M50/1 and XB6B4), *R. hominis* (A2-183) and *E. rectale* (A1-86, M104/1, T1-815 and ATCC33656). **b.** Number of core genes as a function of the number of *Roseburia*/*E. rectale* genomes in pan-genome. A power trendline equation is used to estimate the number of core genes (y) for a given number of genome sequences (x). **c.** Number of new genes (genes with no orthologs in the pan-genome) as a function of the number of *Roseburia*/*E. rectale* genomes in pan-genome. A power trendline equation is used to estimate the number of new genes (y) with the addition of each subsequent genome sequence (x) to the pan-genome. The dramatic increases associated with the addition of new species into the pan-genome have been excluded this plot. Therefore, this equation assumes that no new bacterial species are added to the *Roseburia*/*E. rectale* pan-genome.

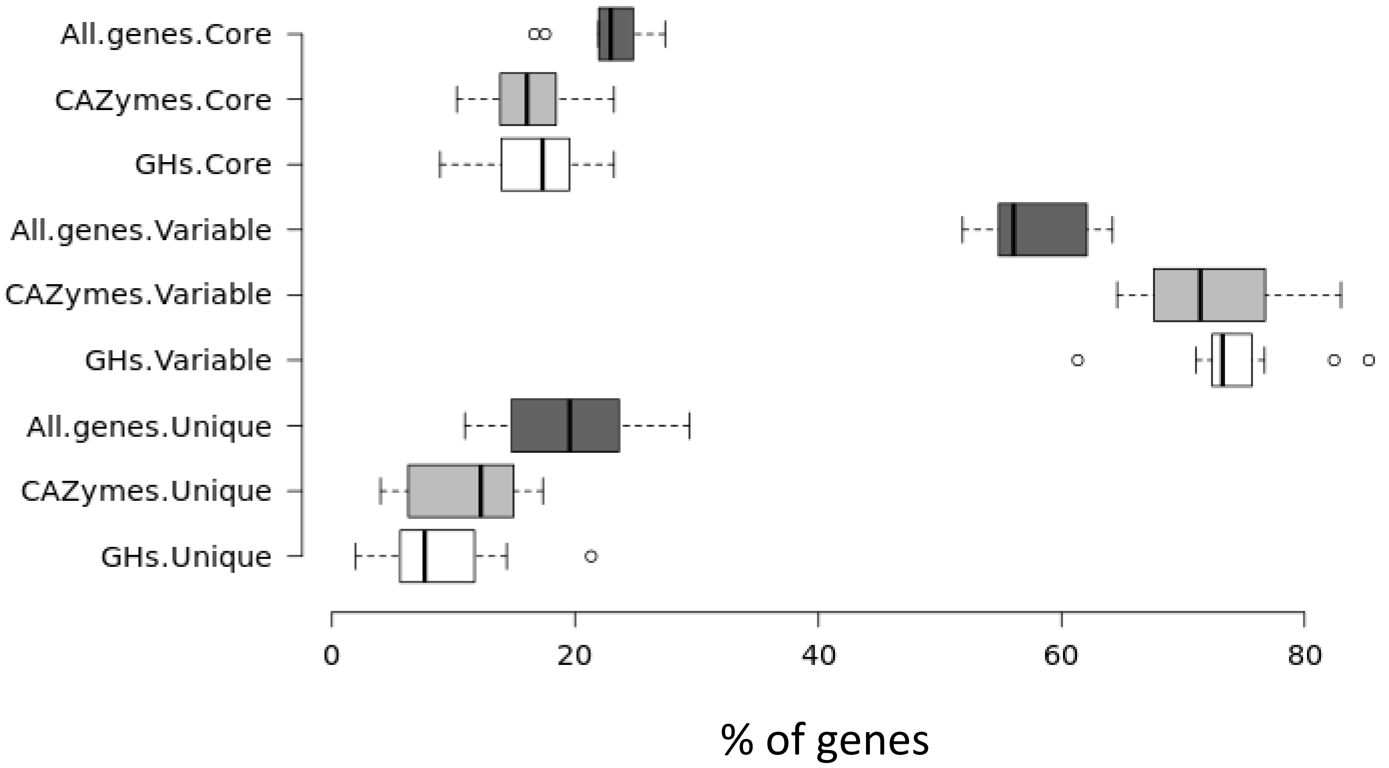

Figure S5. Tukey boxplot of the distribution of GHs, CAZymes and all genes between the core-, variable- and unique-genomes of the *Roseburia*/*E. rectale* pan-genome. All genes (dark gray), CAZymes (light grey) and GHs (white).

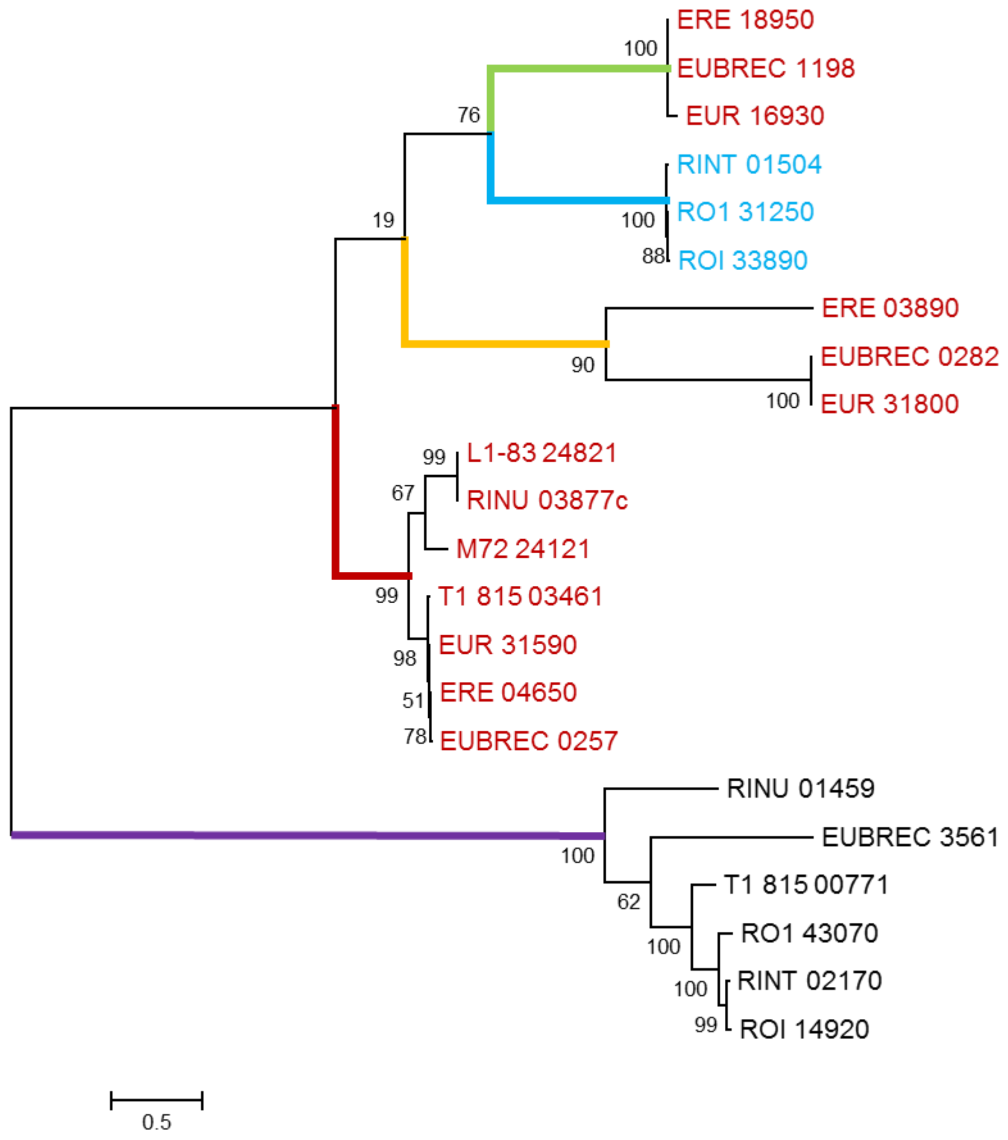

Figure S6. Phylogenetic tree of *Roseburia*/*E. rectale* GH32s. Enzyme names are colour-coded based on KEGG GH annotation. Strongly supported clades (bootstrap ≥ 90) are coloured at their most proximal branch, with each branch colour representing a different clade. Colour-coding of enzymes is as follows: levanase [EC:3.2.1.65] (blue), beta-fructofuranosidase [EC:3.2.1.26] (red) and enzymes with no KEGG GH annotation but that were annotated as GH32 by dbCAN (black). Bootstrap values, expressed as a percentage of 1000 replications, are given at the branching nodes. This tree is unrooted and constructed using the maximum likelihood method. The scale bar refers to the number of amino acid differences per position.

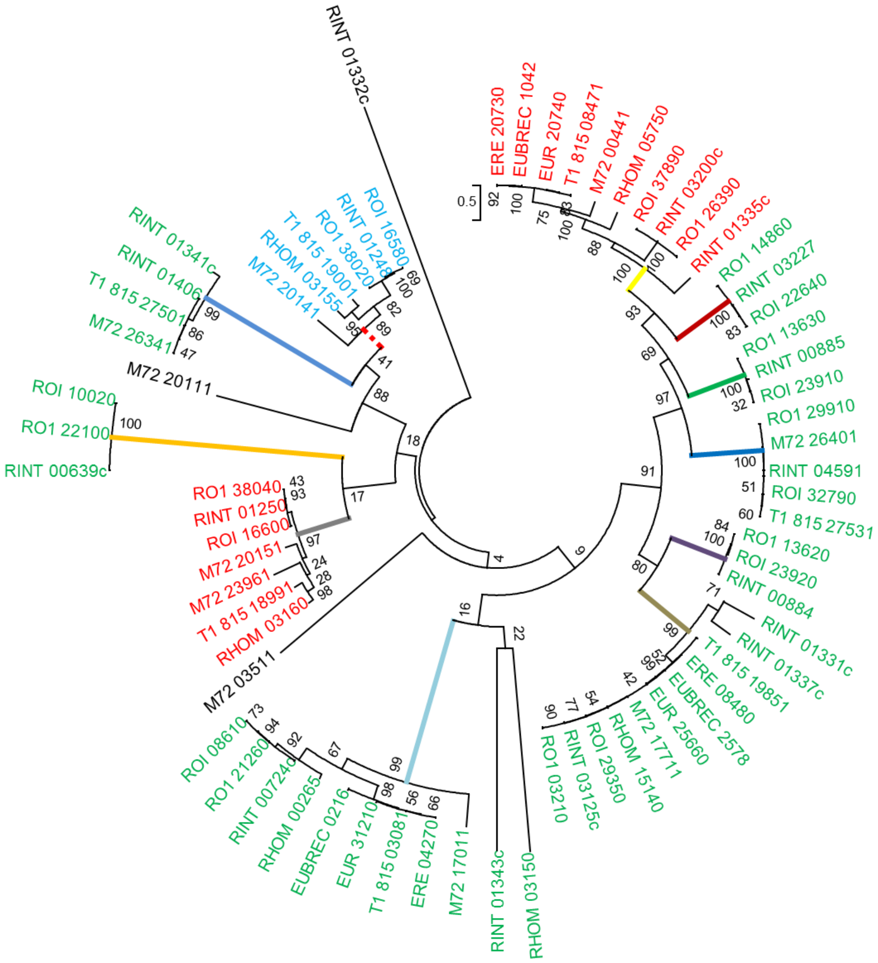

Figure S7. Phylogenetic tree of *Roseburia/E. rectale* GH43s. Enzyme names are colour-coded based on KEGG GH annotation. Strongly supported clades (bootstrap ≥ 90) are coloured at their most proximal branch, with each branch colour representing a different clade. The arabinan endo-1,5-alpha-L-arabinosidase [EC:3.2.1.99] clade (bootstrap 89) is indicated with a red dashed line at its most proximal branch. Colour-coding is as follows: alpha-N-arabinofuranosidase [EC:3.2.1.55] (red), xylan-1,4-beta-xylosidase [EC:3.2.1.37] (green), arabinan endo-1,5-alpha-L-arabinosidase [EC:3.2.1.99] (blue) and enzymes with no KEGG GH annotation (black). Bootstrap values, expressed as a percentage of 1000 replications, are given at the branching nodes. This tree is unrooted and constructed using the maximum likelihood method. The scale bar refers to the number of amino acid differences per position.

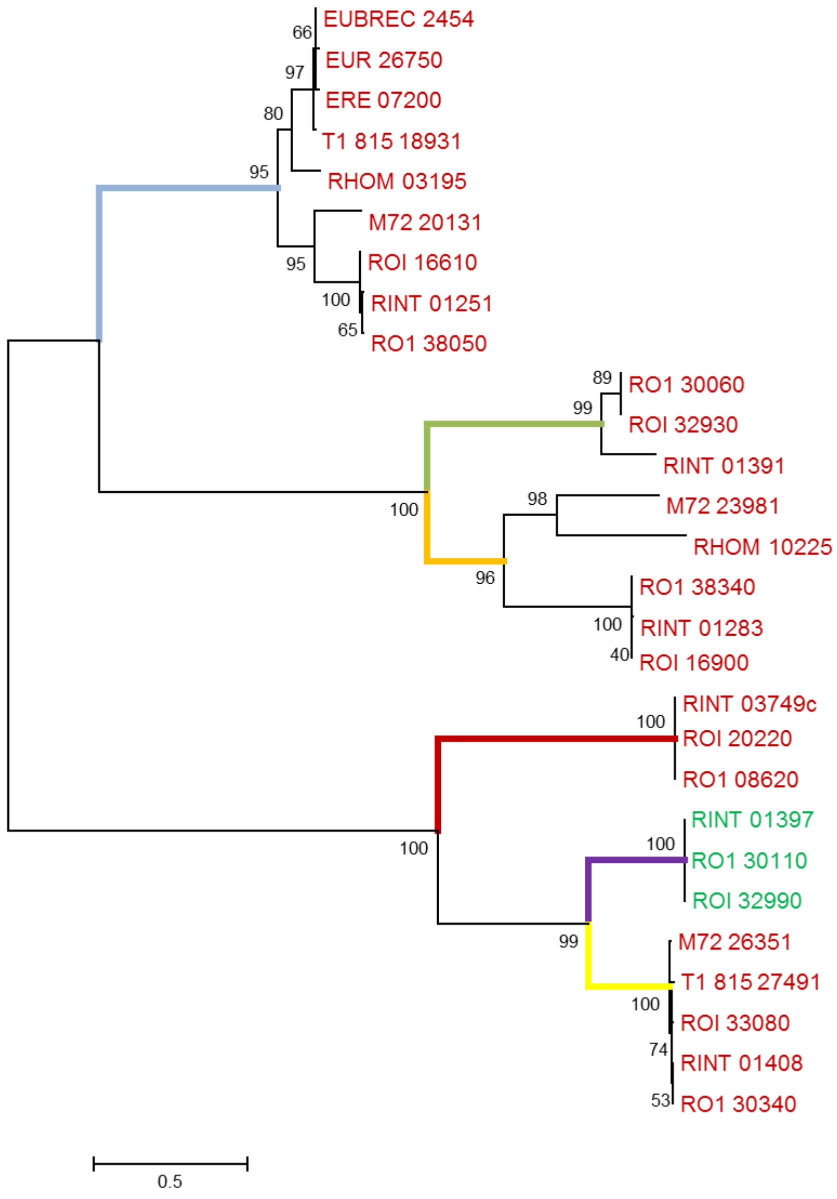

Figure S8. Phylogenetic tree of *Roseburia*/*E. rectale* GH51s. Strongly supported clades (bootstrap ≥ 90) are coloured at their most proximal branch, with each branch colour representing a different clade. Enzyme names are colour-coded based on KEGG GH annotation. Colour-coding is as follows: alpha-N-arabinofuranosidase [EC:3.2.1.55] (red) and xylan-1,4-beta-xylosidase [EC:3.2.1.37] (green). Bootstrap values, expressed as a percentage of 1000 replications, are given at the branching nodes. This tree is unrooted and constructed using the maximum likelihood method. The scale bar refers to the number of amino acid differences per position.

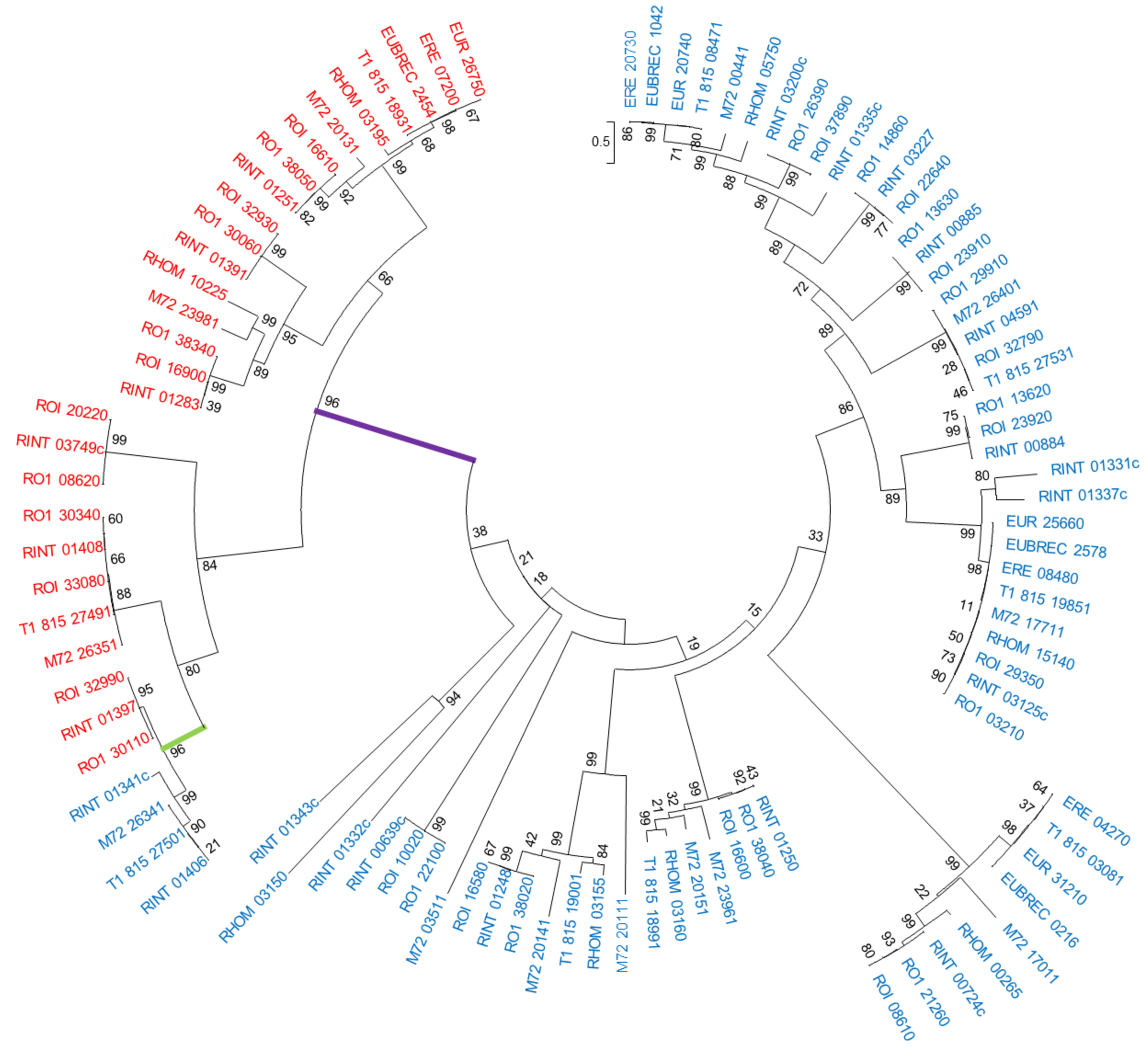

Figure S9. Phylogenetic tree of *Roseburia*/*E. rectale* GH43s and GH51s combined. The GH43s (blue) and GH51s (red) from the *Roseburia*/*E. rectale* genomes in this study were plotted on a single phylogenetic tree. The strongly supported clade (bootstrap ≥ 90) that possesses all GH51s is coloured purple at its most proximal branch. The strongly supported xylan-1,4-beta-xylosidase clade possessing members from both GH43 and GH51 is coloured green at its most proximal branch. Bootstrap values, expressed as a percentage of 1000 replications, are given at the branching nodes. This tree is unrooted and constructed using the maximum likelihood method. The scale bar refers to the number of amino acid differences per position.

a. **b.**

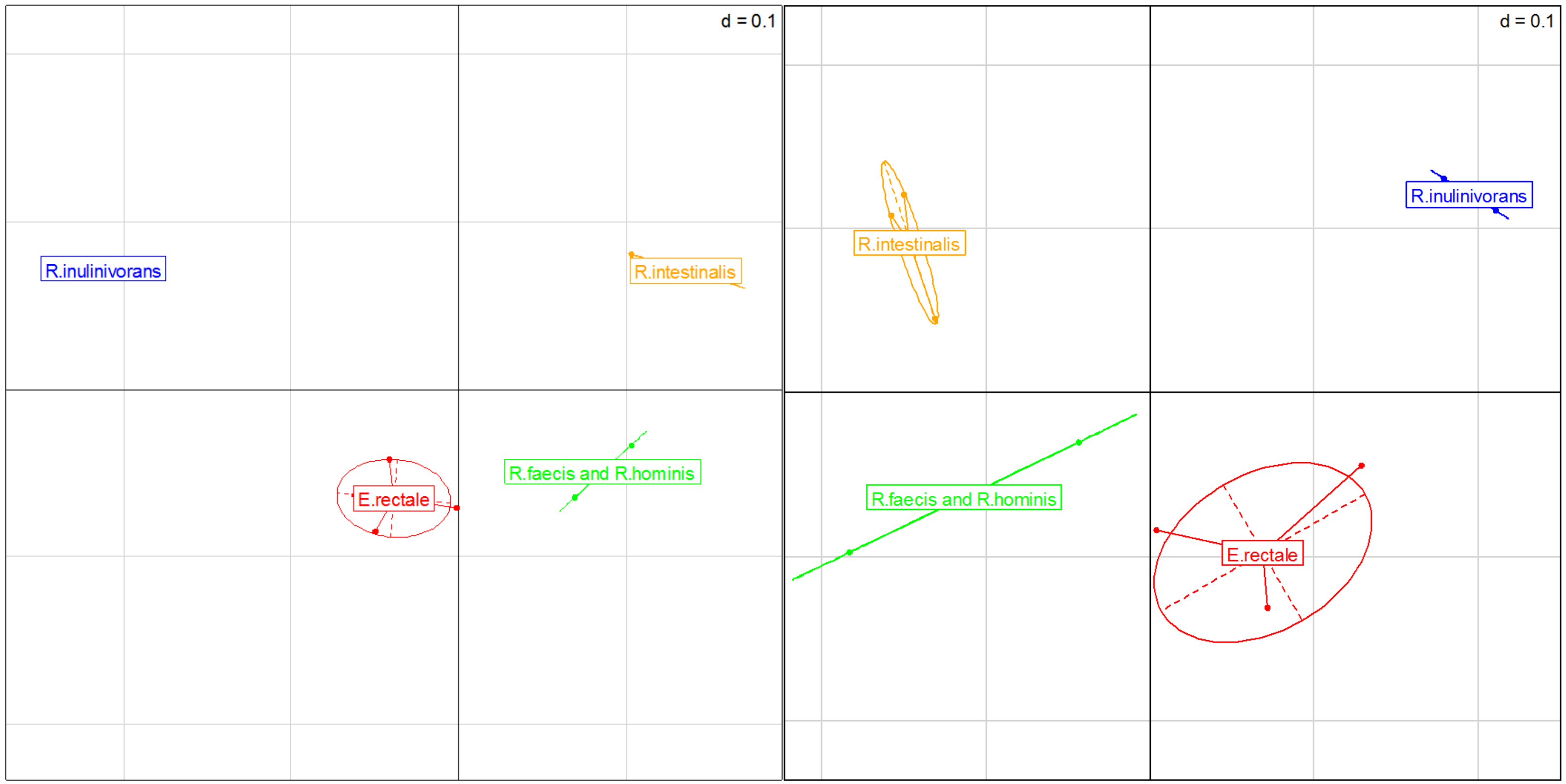

Figure S10. Principal coordinate analysis (PCoA) of *Roseburia*/*E. rectale* strains based on complement of GH families (a) and carbohydrate sets (b). Values of a given GH family or carbohydrate set were taken as the number of these genes each genome possessed. Coordinates were calculated using Kendal tau distance applied to the first five eigenvectors. *R. intestinalis* strains L1-82, M50/1 and XB6B4 (orange); *R. inulinivorans* strains A2-194 and L1-83 (blue); *R. faecis* M72/1 and *R. hominis* A2-183 (green); and *E. rectale* strains A1-86, T1-815, M104/1 and ATCC33656 (red) form separate clusters, both before (a) and after (b) data transformation ($P < 0.001$, Non-parametric MANOVA).

Table S1. List of carbohydrate substrates

Substrate	Supplier	Catalogue number/ Product description
**Fructooligosaccharide (FOS)**	Beneo-Orafti	P95
**Galactooligosaccharide (GOS)**	Friesland Campina Domo	Purified GOS
**Xylooligosaccharide (XOS)**	Shangdong Longlive Biotechnology Co. Ltd	Purified XOS
**Mucin core type 2 (Mucin T2)**	Sigma	M2378
**Mucin core type 3 (Mucin T3)**	Sigma	M1778
**Amylopectin (AP)**	Sigma	S9679
**Amylose (A)**	Sigma	S4180
**Beta-(1,3-1,4)-glucan (β-glu)**	Polycell technologies	Glucagel
**Arabinoxylan (AX)**	Megazyme	High viscosity rye arabinoxylan
**Type 1 arabinogalactan (AG1)**	Megazyme	Galactan (ex. lupin)
**Type 2 arabinogalactan (AG2)**	Sigma	10830
**Xyloglucan (XG)**	Megazyme	(amyloid) from tamarind seed
**Inulin (I)**	Sigma	I3754
**Beta-mannan (β-man)**	Megazyme	Ivory nut mannan

Table S2. GHs represented in each carbohydrate set

Carbohydrate sets	Specific GH families (GH)	EC numbers (EC:3.2.1.X)
**Pectins**	28, 78, 105	15, 40
**Beta-glucans**	55,74, 94	4, 21, 39, 86
**Host-derived**	29, 76, 84, 85, 98, 109, 129	24, 45, 49, 51
**Xylans and arabinans**	10, 120	8, 37, 55, 99, 131
**Beta-mannans**	113, 125, 130	25, 78
**Alpha- and beta-galactosides**		22, 23
**Alpha-glucans**	13, 15, 77	3, 8*, 20, 41
**Fructans**	32	
**Type-1 arabinogalactan**	53	

"Specific GH families" are predicted to exclusively target carbohydrates of one set. Polyspecificity in GH families was resolved using EC numbers predicted by KEGG GH. *2.4.1.8 not 3.2.1.8.

Table S3. Maximum growth (OD_{650}) achieved by *Roseburia*/*E. rectale* strains during growth on dietary and host-derived carbohydrates.

Species	Strain	FOS	GOS	XOS	AP	A	β-glu	AX	AG 1	I
E. rectale	**A1-86**	0.52 ± 0.02	-	0.79 ± 0.01	1.14 ± 0.04	0.52 ± 0.07	-	0.53 ± 0.11	-	0.18 ± 0.03
	**M104/1**	1.10 ± 0.02	0.95 ± 0.02	0.72 ± 0.01	1.2 ± 0.06	0.41 ± 0.04	-	-	-	0.45 ± 0.09
	**T1-815**	1.18 ± 0.02	0.75 ± 0.02	0.84 ± 0.02	1.14 ± 0.04	0.6 ± 0.02	-	0.73 ± 0.09	-	0.30 ± 0.03
R. inulinivorans	**A2-194**	0.58 ± 0.01	0.45 ± 0.01	-	1.18 ± 0.02	0.56 ± 0.02	-	-	-	0.39 ± 0.004
	**L1-83**	0.56 ± 0.02	-	-	0.54 ± 0.02	0.38 ± 0.05	0.44 ± 0.11	-	-	0.27 ± 0.01
R. intestinalis	**L1-82**	1.27 ± 0.02	0.64 ± 0.04	0.85 ± 0.02	-	0.74 ± 0.07	-	1.01 ± 0.09	-	-
	**M50/1**	1.29 ± 0.03	0.77 ± 0.02	0.82 ± 0.03	1.64 ± 0.02	0.4 ± 0.07	-	0.54 ± 0.07	-	-
	**XB6B4**	1.21 ± 0.02	0.76 ± 0.02	0.87 ± 0.03	1.21 ± 0.05	-	-	0.5 ± 0.05	-	-
R. hominis	**A2-183**	0.85 ± 0.02	-	0.73 ± 0.02	-	-	-	-	-	-
R. faecis	**M72/1**	1.00 ± 0.01	0.76 ± 0.004	0.64 ± 0.01	1.09 ± 0.08	0.34 ± 0.10	0.8 ± 0.37	0.36 ± 0.1	1.08 ± 0.04	-

Growth was performed in microtitre plates on all substrates with the exception of inulin, beta-mannan and mucin, where growth was measured by optical density in Hungate tubes and confirmed by gas production. Hyphens (-) represent no growth. Data presented here are the average maximum OD_{650} readings of six replicates ± standard deviation for microtitre plate experiments and three replicates ± standard deviation for Hungate tube experiments. No growth was observed for any the eleven strains on beta-mannan, xyloglucan, type 2 arabinogalactan, mucin core-type 2 or mucin core-type 3. Fructooligosaccharide (**FOS**), galactooligosaccharide (**GOS**), xylooligosaccharide (**XOS**), amylopectin (**AP**), amylose (**A**), beta-(1,3-1,4)-glucan (**β-glu**), arabinoxylan (**AX**), type 1 arabinogalactan (**AG1**) and inulin

(**I**). Previous reports that *R. intestinalis* is able to use starch as a growth substrate may be due to differences in the substrate preparation method or amylopectin content.

Table S4. ***Roseburia/E. rectale*** **genome information**

**Species**	**Strain**	**Genome accession No.**	**No. ORFs**	**No. Contigs**	**Contig N50 (nt)**	**No. Scaffolds**	**Scaffold N50 (nt)**	**Size (nt)**	**Genome Publication Reference**
E. rectale	ATCC33656	NC_012781.1	3621	Complete Genome	n/a	1	n/a	3,449,685	Unpublished
	A1-86	NC_021010.1	2898	90	80,495	n/a	n/a	3,344,951	Unpublished
	M104/1	NC_021044.1	3206	128	62,843	n/a	n/a	3,698,419	Unpublished
	T1-815	CVRQ01000001-CVRQ01000090	2896	89	124,756	n/a	n/a	3,045,135	This work
R. inulinivorans	A2-194	ACFY01000000	4522	179	57,343	n/a	n/a	4,048,462	Unpublished
	L1-83	CVRS01000001-CVRS01000151	3488	152	70,539	n/a	n/a	3,781,521	This work
R. intestinalis	L1-82	ABYJ00000000.2	4766	409	29,464	102	123,125	4,411,375	Unpublished
	M50/1	NC_021040.1	3461	144	57,879	n/a	n/a	4,143,550	Unpublished
	XB6B4	NC_021012	3610	154	87,898	n/a	n/a	4,286,292	Unpublished
R. hominis	A2-183	CP003040.1	3362	Complete Genome	n/a	1	n/a	3,592,125	Unpublished
R. faecis	M72/1	CVRR01000001-CVRR01000101	3205	101	143,514	n/a	n/a	3,334,694	This work

Number of (No.) and Open reading frame (ORF). N50 is a statistical method used to indicate the quality of a sequence. Sequencing and genome assembly of *E. rectale* T1-815, *R. inulinivorans* L1-83 and *R. faecis* M72/1 was performed by the Wellcome Trust Sanger Institute, Hinxton, UK

Table S5. The numbers of glycoside hydrolase (GH) family representatives in the *Roseburia*/*E. rectale* group genomes.

GH family:	1	2	3	4	5	8	10	13	15	16	18	19	20	22	23	24	25	26	27	28	29	30	31	32	35	36	38	39	42	43	51	53	55
***E. rectale* A1-86**	1	3	4	0	1	1	0	13	0	0	1	0	0	0	2	0	1	0	0	0	0	0	3	2	0	1	0	0	2	2	1	3	0
***E. rectale* ATCC33656**	1	3	3	1	1	1	0	13	0	1	1	0	0	0	1	1	5	0	0	0	0	0	3	3	0	3	0	0	2	2	1	3	0
***E. rectale* M104/1**	1	4	3	0	1	1	0	13	0	1	1	0	0	1	1	0	1	0	0	0	0	0	2	2	0	1	0	0	2	2	1	3	0
***E. rectale* T1-815**	1	3	5	1	1	1	1	12	0	1	1	0	0	0	3	0	3	0	0	1	0	0	2	2	0	1	0	0	2	6	2	5	0
***R. faecis* M72/1**	1	4	3	1	1	1	1	12	0	1	1	0	0	0	2	0	3	1	1	0	0	0	1	1	0	4	0	1	2	9	3	7	0
***R. hominis* A2-183**	3	6	5	2	0	1	0	8	1	0	1	0	0	0	1	0	0	0	1	2	0	0	1	0	0	2	0	0	1	5	2	7	0
***R. intestinalis* L1-82**	2	9	14	3	1	1	2	13	0	0	1	0	1	0	3	2	1	1	2	1	1	0	4	2	1	3	3	1	2	16	6	4	0
***R. intestinalis* M50/1**	1	7	10	1	0	1	0	12	0	0	1	0	0	0	3	0	0	1	1	1	1	0	4	2	1	3	1	1	2	9	6	3	0
***R. intestinalis* XB6B4**	1	8	18	3	1	1	1	12	0	0	1	0	1	0	2	0	1	1	1	1	1	0	4	2	2	3	3	2	2	9	6	2	0
***R. inulinivorans* A2-194**	1	5	12	0	1	0	0	10	0	0	1	1	1	0	0	0	2	0	0	0	2	1	2	2	0	2	0	0	2	0	0	1	1
***R. inulinivorans* L1-83**	1	4	10	1	1	0	0	12	0	1	1	0	1	0	0	0	2	0	0	1	2	1	1	1	0	2	0	0	1	0	0	1	0

GH family:	63	65	73	74	76	77	78	79	84	85	88	94	95	98	105	109	112	113	114	115	120	125	127	129	130	NC*	Total**
***E. rectale* A1-86**	0	0	1	0	0	1	1	0	1	0	0	3	0	0	0	2	1	0	0	0	0	0	0	0	0	5	56
***E. rectale* ATCC33656**	0	0	0	0	0	3	1	0	1	0	0	3	0	0	0	5	1	0	0	0	0	0	0	0	0	7	70
***E. rectale* M104/1**	0	0	0	0	0	1	1	0	1	0	0	3	0	0	0	2	1	0	0	1	0	0	0	0	0	6	57
***E. rectale* T1-815**	0	0	0	0	0	2	1	0	0	0	0	1	0	0	0	4	1	0	0	0	0	0	1	0	0	3	67
***R. faecis* M72/1**	0	0	0	0	1	2	1	0	1	0	0	3	0	0	0	4	1	1	1	1	1	0	1	0	2	5	86
***R. hominis* A2-183**	0	0	1	0	1	1	2	1	1	0	1	5	0	0	1	3	1	1	0	1	0	0	1	0	2	4	76
***R. intestinalis* L1-82**	0	1	1	2	1	2	3	0	1	1	1	4	1	0	2	2	1	1	1	2	1	1	3	1	4	9	146
***R. intestinalis* M50/1**	0	1	0	1	1	2	2	0	1	0	1	3	1	0	1	2	1	1	1	0	1	0	2	0	2	6	102
***R. intestinalis* XB6B4**	1	0	0	1	1	2	5	0	1	1	1	4	0	0	1	2	1	1	1	1	2	1	2	0	4	9	131
***R. inulinivorans* A2-194**	0	0	1	0	0	4	1	0	1	0	0	3	2	1	1	2	1	0	1	0	0	0	0	0	0	10	75
***R. inulinivorans* L1-83**	0	1	0	0	0	2	1	0	1	0	0	3	2	0	1	3	1	0	1	0	0	0	0	0	0	6	66

*GHs annotated solely by BLASTp query against KEGG GH could not be assigned into families and are presented here as non-classified (NC).
**For comparison, the total number of GH domains detected by www.cazy.org: A1-86 (47), ATCC33656 (52), M104/1 (46), A2-183 (63) and XB6B4 (114).

Table S6. The numbers of glycosyltransferase (GT), carbohydrate esterase and polysaccharide lyase family representatives in the *Roseburia*/*E. rectale* group genomes.

GT family:	2	4	5	8	11	12	13	14	19	23	26	27	28	30	32	35	39	46	51	78	83	90
***E. rectale* A1-86**	15	9	2	1	0	0	0	0	0	0	1	0	1	1	0	2	1	0	2	0	1	0
***E. rectale* ATCC33656**	13	10	2	1	0	0	0	0	0	0	1	0	6	1	0	2	1	0	3	0	1	0
***E. rectale* M104/1**	14	6	2	1	0	0	0	0	0	0	1	0	1	1	0	2	1	0	2	0	1	0
***E. rectale* T1-815**	30	10	2	0	1	1	0	0	0	1	1	0	4	1	3	2	1	0	2	0	1	0
***R. faecis* M72/1**	10	3	2	2	0	0	0	0	0	0	1	0	2	0	0	2	1	0	2	0	2	1
***R. hominis* A2-183**	24	7	2	4	1	0	1	0	1	2	2	0	4	0	0	2	1	0	2	0	3	0
***R. intestinalis* L1-82**	24	7	2	0	1	0	1	0	1	1	2	0	4	1	0	2	1	1	3	0	2	0
***R. intestinalis* M50/1**	25	6	2	3	1	1	1	0	1	1	2	0	3	1	0	2	1	0	2	0	0	0
***R. intestinalis* XB6B4**	16	5	2	2	1	0	1	0	1	1	2	0	3	1	0	2	1	0	2	1	0	0
***R. inulinivorans* A2-194**	24	6	3	0	0	0	0	0	1	0	1	1	4	1	1	2	0	0	2	1	1	0
***R. inulinivorans* L1-83**	29	9	2	0	0	0	0	1	2	0	1	1	4	1	0	2	0	0	2	1	1	0

CAZyme family:	CE1	CE2	CE3	CE4	CE6	CE7	CE8	CE9	CE12	CE14	PL12
***E. rectale* A1-86**	2	0	0	3	0	2	0	2	0	0	0
***E. rectale* ATCC33656**	3	0	0	3	0	2	0	2	3	0	0
***E. rectale* M104/1**	4	0	0	4	1	2	0	2	0	0	0
***E. rectale* T1-815**	5	1	0	3	0	2	0	2	0	0	0
***R. faecis* M72/1**	2	1	1	4	0	0	0	2	0	1	0
***R. hominis* A2-183**	4	1	6	4	0	1	1	2	0	0	1
***R. intestinalis* L1-82**	11	1	5	6	0	1	1	2	1	0	0
***R. intestinalis* M50/1**	9	1	5	4	0	1	1	2	1	0	0
***R. intestinalis* XB6B4**	9	1	4	5	0	1	1	3	1	0	0
***R. inulinivorans* A2-194**	4	1	4	3	0	2	1	3	0	1	0
***R. inulinivorans* L1-83**	10	2	4	3	0	1	1	3	0	1	0

Carbohydrate esterase (CE) and polysaccharide lyase (PL)

Table S7. Signal peptide possessing glycoside hydrolases of the *Roseburia*/*E. rectale* group

KEGG GH	dbCAN	TMH	*E. rectale* A1-86	*E. rectale* ATCC33656	*E. rectale* M104/1	*E. rectale* T1-815	*R. faecis* M72/1	*R. hominis* A2-183	*R. intestinalis* L1-82	*R. intestinalis* M50/1	*R. intestinalis* XB6B4	*R. inulinivorans* A2-194	*R. inulinivorans* L1-83
alpha- amylase_[EC:3.2.1.1]	GH13	1	EUR_01860	EUBREC_0546	ERE_24130	T1_815_06021	M72_10591	RHOM_12770	RINT_03907	ROI_03530	RO1_25110	RINU_01533	L1-83_20691
arabinogalactan endo-1,4-beta-galactosidase [EC:3.2.1.89]	GH53 + CBM61	1	-	-	-	T1_815_06031	M72_01461	RHOM_06170	RINT_02688c	-	-	-	-
arabinogalactan endo-1,4-beta-galactosidase [EC:3.2.1.89]	GH53 + CBM61	0	-	-	-	-	-	-	RINT_01496	ROI_33860	-	-	-
arabinogalactan endo-1,4-beta-galactosidase [EC:3.2.1.89]	CBM61	0	-	-	-	-	M72_25701	RHOM_10760	-	-	-	-	-
arabinogalactan endo-1,4-beta-galactosidase [EC:3.2.1.89]	GH53	0	-	-	-	-	-	RHOM_10735	-	-	-	-	-
beta-1,3-glucanase [EC:3.2.1.39]	4 X CBM37	0	-	-	-	-	-	-	-	ROI_16540	-	-	-
beta-xylosidase [EC:3.2.1.99]	GH43	0	-	-	-	T1_815_19001	M72_20141	RHOM_03155	RINT_01248	ROI_16580	RO1_38020	-	-
cyclomaltodextrinase [EC:3.2.1.4]	GH13	0	-	-	-	-	-	-	RINT_03777c	ROI_28570	RO1_38940	-	-
endo-1,4-beta-xylanase [EC:3.2.1.8]	GH10 + 2 X CBM9	1	-	-	-	T1_815_08451	M72_00471	-	RINT_01499	-	RO1_31190	-	-
endoglucanase [EC: 3.2.1.4]	GH5	0	-	-	-	-	-	-	RINT_03566c	-	RO1_39790	-	-
endoglucanase [EC:3.2.1.4]	*	1	-	-	-	-	M72_11231	-	-	-	-	-	-
endoglucanase [EC:3.2.1.4]	*	1	-	-	-	-	-	RHOM_00990	-	-	-	-	-
endoglucanase_[EC:3.2.1.4]	GH5	1	EUR_02280	EUBREC_0616	ERE_23730	T1_815_06231	M72_10311	-	-	-	-	RINU_02254	L1-83_07051
glucan endo-1,3-D-glucosidase [EC:3.2.1.39]	GH16 + 4 X CBM4	0	-	-	-	-	M72_15771	-	-	-	-	-	L1-83_07221
lysozyme [EC:3.2.1.17]	GH25	1	EUR_05130	EUBREC_3299	ERE_14340	T1_815_24911	M72_23791	-	-	-	-	RINU_04118c	L1-83_09151
lysozyme [EC:3.2.1.17]	GH25 + CBM37	0	-	-	-	-	M72_27751	-	-	-	-	-	-
mannan endo-1,4-betamannosidase [EC:3.2.1.78]	GH53 + 2 X CBM13 + CBM65	1	-	-	-	-	M72_19931	-	-	-	-	-	-
mannan endo-1,4-beta-mannosidase [EC:3.2.1.78]	GH26 + CBM27 + CBM23	0	-	-	-	-	M72_25771	-	RINT_02694c	ROI_01180	RO1_41120	-	-
mannosyl glycoprotein endo-beta-N-acetylglucosaminidase [EC:3.2.1.96]	GH85 + CBM32	0	-	-	-	-	-	-	RINT_01675c	-	RO1_41310	-	-
mannosyl-glycoprotein endo-beta-N-acetylglucosaminidase [EC:3.2.1.96]	2 X CBM13	1	EUR_11650	-	-	-	-	-	-	-	-	-	-
pullulanase [EC:3.2.1.41]	GH13 + 2 X CBM26	2	EUR_21100	EUBREC_1081	ERE_20420	T1_815_08821	M72_12731	-	-	-	-	RINU_03380	L1-83_29381
pullulanase [EC:3.2.1.41]	CBM37	0	-	-	ERE_36420	-	-	-	RINT_01948	ROI_34260	-	-	-
putative lipoprotein [EC:3.2.1.14]	GH23	1	-	-	-	T1_815_26381	M72_07731	-	RINT_02000c	ROI_11590	-	-	L1-83_01111
	GH114	1	-	-	-	-	M72_19981	-	RINT_02832c	ROI_13730	RO1_02530	RINU_02200	L1-83_13751
	GH23	1	EUR_29710	-	-	T1_815_23241	-	-	-	-	-	-	-
	GH25	0	-	EUBREC_2636	-	-	-	-	-	-	-	-	-
	GH25 + 3 X CBM37	0	-	-	-	-	M72_01971	-	-	-	-	-	-
	GH25 + 5 X CBM37	0	-	-	-	-	-	-	RINT_01245	-	RO1_37990	-	-
	GH3	2	-	-	-	-	-	-	-	-	-	-	L1-83_30771
	GH4	1	-	-	-	-	-	-	-	-	RO1_21680	-	-
	GH73	0	-	-	-	-	-	RHOM_12165	-	-	-	-	-
	GH98 + 2 X CBM51	1	-	-	-	-	-	-	-	-	-	RINU_02230c	-

Horizontal cells are members of the same ortholog group (OG). The 'KEGG GH' and 'dbCAN' columns show the annotation conferred upon the OG by these databases. The 'TMH' column shows the number of transmembrane helices in the OG predicted by TMHMM Server v. 2.0 (http://www.cbs.dtu.dk/services/TMHMM/). Purple boxes indicate proteins that are not predicted to have signal peptides, but share an OG with proteins that do. Pink boxes indicate proteins that are predicted to possess a number of transmembrane helices that is different from that of the majority of proteins in the OG. Green boxes indicate proteins that are not predicted to have signal peptides, but share an OG with proteins that do and also possess a number of transmembrane helices that is different from that of the majority of proteins in the OG. Glycoside hydrolase (GH) and carbohydrate binding module (CBM). *CAZyme conserved domains could not be detected in M72_11231 and RHOM_00990, making their prediction as glycoside hydrolases more tenuous than the others in this table.

Table S8. Carbohydrate binding module (CBM) possessing glycoside hydrolases (GHs) of the *Roseburia*/*E. rectale* group

KEGG GH	dbCAN	Erectale_A1_8	Erectale_ATCC336	Erectale_M104_	Erectale_T1_81	R.faecis_M72_	Rhominis_A2_18	Rintestinalis_L1_8	Rintestinalis_M50_	Rintestinalis_XB6B	Rinulinivorans_A2_19	R.inulinivorans_L1_8
endoglucanase [EC: 3.2.1.4]/chitinase [EC:3.2.1.14]	2 x CBM2	-	EUBREC_0268	ERE_12690	-	M72_15351	-	-	-	-	-	-
pullulanase [EC:3.2.1.41]	CBM37	-	-	ERE_36420	-	-	-	RINT_01948	ROI_34260	-	-	-
1,4-beta-N-acetylmuramidase	CBM50	-	-	-	-	-	RHOM_02240	RINT_01150	ROI_15650	RO1_36820	RINU_01480	L1-83_20261
xylan-1,4-beta-xylosidase [EC:3.2.1.37]	CBM6	-	-	-	-	-	-	RINT_01340c	-	-	-	-
endo-1,4-beta-xylanase [EC:3.2.1.8]	GH10 + CBM22	-	-	-	-	-	-	RINT_01338c	-	-	-	-
	GH115 + CBM35	-	-	-	-	M72_00501	RHOM_05795	RINT_00115	-	-	-	-
	GH115 + CBM35	-	-	-	-	-	-	RINT_03202c	-	RO1_26370	-	-
alpha-glucosidase [EC:3.2.1.3]	GH13 + CBM34	-	-	-	-	-	-	RINT_01191	ROI_16030	RO1_37230	RINU_01526	L1-83_20631
neopullulanase [EC: 3.2.1.135]	GH13 + CBM34	EUR_14770	-	ERE_28930	T1_815_14701	M72_09701	RHOM_03535	-	-	-	-	-
neopullulanase [EC:3.2.1.135]	GH13 + CBM34	EUR_05180	EUBREC_3295	ERE_14300	T1_815_24901	M72_26561	-	RINT_01429	ROI_33260	RO1_30550	RINU_04311	L1-83_22111
cyclomaltodextrinase	GH13 + CBM48	-	EUBREC_1186	-	-	-	RHOM_00205	-	-	-	-	-
glycogen_debranching_enzyme_Glg X_[EC:3.2.1.-]	GH13 + CBM48	EUR_08140	EUBREC_2964	ERE_11570	T1_815_22431	M72_03121	RHOM_14780	RINT_02827c	ROI_13780	RO1_02480	RINU_02055	L1-83_32271
pullulanase [EC: 3.2.1.41]	GH13 + CBM48	EUR_14250	EUBREC_1843	ERE_28440	T1_815_14451	M72_18671	-	RINT_00448c	ROI_37050	RO1_25680	RINU_01524c	L1-83_20621
maltooligosyltrehalose_ trehalohydrolase_[EC:3.2.1.141]	GH13 + CBM48	EUR_28650	EUBREC_0027	ERE_01850	T1_815_00191	M72_19881	RHOM_15865	RINT_00264c	ROI_34590	RO1_31890	RINU_02213c	L1-83_28751
beta-galactosidase [EC:3.2.1.23]	GH2 + 2 X CBM6	-	-	-	-	-	-	RINT_01325	-	-	-	-
lysozyme [EC:3.2.1.17]	GH24 + 3 X CBM37	-	EUBREC_2755	-	-	-	-	-	-	-	-	-
beta-fructofuranosidase [EC:3.2.1.26]	GH32 + CBM66	EUR_31800	EUBREC_0282	ERE_03890	-	-	-	-	-	-	-	-
xylan-1,4-beta-xylosidase [EC:3.2.1.37]	GH43 + CBM42	-	-	-	-	-	RHOM_03150	-	-	-	-	-
xylan-1,4-beta-xylosidase [EC:3.2.1.37]	GH43 + CBM6	-	-	-	T1_815_27501	M72_26341	-	RINT_01406	-	-	-	-
xylosidase/arabinofuranosidase [EC:3.2.1.37]	GH43 + CBM6	-	-	-	-	-	-	RINT_01343c	-	-	-	-
alpha-N-arabinofuranosidase [EC:3.2.1.55]	GH51 + CBM4	-	-	-	-	-	-	RINT_03749c	ROI_20220	RO1_08620	-	-
xylan-1,4-beta-xylosidase [EC:3.2.1.37]	GH51 + GH43 + CBM6	-	-	-	-	-	-	RINT_01397	ROI_32990	RO1_30110	-	-
arabinogalactan endo-1,4-beta-galactosidase (EC:3.2.1.89)	GH53 + CBM16	EUR_20400	EUBREC_1006	ERE_21070	T1_815_08171	M72_21201	RHOM_13045	RINT_03592c	ROI_27920	RO1_39580	-	-
	GH53 + CBM16	EUR_20040	EUBREC_0970	ERE_21440	T1_815_07881	-	-	-	-	-	-	-
arabinogalactan endo-1,4-beta-galactosidase [EC:3.2.1.89]	GH53 + CBM61	-	-	-	-	M72_25751	RHOM_10730	-	-	-	-	-
alpha-L-rhamnosidase [EC:3.2.1.40]	GH78 + CBM67	-	-	-	-	-	RHOM_10660	RINT_04581c	-	RO1_07080	-	-
alpha-L-rhamnosidase [EC:3.2.1.40]	GH78 + CBM67	-	-	-	-	-	-	-	-	RO1_35930	-	-
	GH78 + CBM67	-	-	-	-	-	RHOM_04080	RINT_00655c	ROI_09910	RO1_21990	-	-
mannosyl glycoprotein endo-beta-N-acetylglucosaminidase [EC:3.2.1.96]	GH85 + CBM32	-	-	-	-	-	-	RINT_01675c	-	RO1_41310	-	-
mannosyl-glycoprotein endo-beta-N-acetylglucosaminidase [EC:3.2.1.96]	2 X CBM13	EUR_11650	-	-	-	-	-	-	-	-	-	-
beta-1,3-glucanase [EC:3.2.1.39]	4 X CBM37	-	-	-	-	-	-	-	ROI_16540	-	-	-
arabinogalactan endo-1,4-beta-galactosidase [EC:3.2.1.89]	CBM61	-	-	-	-	M72_25701	RHOM_10760	-	-	-	-	-
endo-1,4-beta-xylanase [EC:3.2.1.8]	GH10 + 2 X CBM9	-	-	-	T1_815_08451	M72_00471	-	RINT_01499	-	RO1_31190	-	-
pullulanase [EC:3.2.1.41]	GH13 + 2 X CBM26	EUR_21100	EUBREC_1081	ERE_20420	T1_815_08821	M72_12731	-	-	-	-	RINU_03380	L1-83_29381
glucan endo-1,3-D-glucosidase [EC:3.2.1.39]	GH16 + 4 X CBM4	-	-	-	-	M72_15771	-	-	-	-	-	L1-83_07221
	GH25 + 3 X CBM37	-	-	-	-	M72_01971	-	-	-	-	-	-
	GH25 + 5 X CBM37	-	-	-	-	-	-	RINT_01245	-	RO1_37990	-	-
lysozyme [EC:3.2.1.17]	GH25 + CBM37	-	-	-	-	M72_27751	-	-	-	-	-	-
mannan endo-1,4-beta-mannosidase [EC:3.2.1.78]	GH26 + CBM27 + CBM23	-	-	-	-	M72_25771	-	RINT_02694c	ROI_01180	RO1_41120	-	-
mannan endo-1,4-betamannosidase [EC:3.2.1.78]	GH53 + 2 X CBM13 + CBM	-	-	-	-	M72_19931	-	-	-	-	-	-
arabinogalactan endo-1,4-beta-galactosidase (EC:3.2.1.89)	GH53 + CBM61	-	-	-	T1_815_06031	M72_01461	RHOM_06170	RINT_02688c	-	-	-	-
arabinogalactan endo-1,4-beta-galactosidase (EC:3.2.1.89)	GH53 + CBM61	-	-	-	-	-	-	RINT_01496	ROI_33860	-	-	-
	GH98 + 2 X CBM51	-	-	-	-	-	-	-	-	-	RINU_02230c	-

Horizontal cells are members of the same ortholog group (OG). The ‘KEGG GH’ and ‘dbCAN’ columns show the annotation conferred upon the OG by these databases.

Table S9. Core GHs and conserved species-specific GHs.

Species	OG	KEGG GH	HMM GH
**All**	QTS_29	chitinase [EC:3.2.1.14]	GH18
	QTS_52	Arabinogalactan endo-1,4-beta-galactosidase [EC:3.2.1.89]	GH53
	QTS_139	beta–galactosidase [EC:3.2.1.23]	GH2
	QTS_262		GH77
	QTS_265	alpha-amylase [EC:3.2.1.1]	GH13
	QTS_272		GH112
	QTS_278	alpha-glucosidase [EC:3.2.1.20]	GH31
	QTS_317		GH109
	QTS_459		GH94
	QTS_506	oligo-1,6-glucosidase [EC:3.2.1.10]	GH13
	QTS_561	glycogen debranching enzyme GlgX [EC:3.2.1.-]	GH13 + CBM48
	QTS_692	alpha-amylase Amy13C [EC: 3.2.1.1]	GH13
	QTS_705	maltooligosyltrehalose trehalohydrolase [EC:3.2.1.141]	GH13 + CBM48
R. intestinalis	QTS_3738		3 X GH74
	QTS_4461		GH120
	QTS_4053	oligo-1,6-glucosidase [EC:3.2.1.10]	GH13
	QTS_4482	cyclomaltodextrinase [EC:3.2.1.4]	GH13
	QTS_3883	beta-galactosidase [EC:3.2.1.23]	GH2
	QTS_3589	murein lytic transglycosylase [EC:3.2.1.-]	GH23
	QTS_3868	beta-glucosidase [EC:3.2.1.21]	GH3
	QTS_4115	beta-glucosidase [EC:3.2.1.21]	GH3
	QTS_3434	GH31	GH31
	QTS_4025	levanase [EC:3.2.1.65]	GH32
	QTS_4493	beta-galactosidase [EC:3.2.1.23]	GH35
	QTS_3560	alpha-mannosidase [EC:3.2.1.24]	GH38
	QTS_3467	xylosidase/arabinofuranosidase [EC:3.2.1.37]	GH43
	QTS_3480	xylan-1,4-beta-xylosidase [EC:3.2.1.37]	GH43
	QTS_3631	beta-xylosidase [EC:3.2.1.99]	GH43
	QTS_4414	xylan-1,4-beta-xylosidase [EC:3.2.1.37]	GH43
	QTS_4018	alpha-L-arabinofuranosidase [EC:3.2.1.55]	GH51
	QTS_3865	alpha-L-arabinofuranosidase [EC:3.2.1.55]	GH51 + CBM4
	QTS_4169	xylan-1,4-beta-xylosidase [EC:3.2.1.37]	GH51 + GH43 + CBM6

R. inulinivorans	QTS_5260	lysozyme [EC:3.2.1.17]	GH25
	QTS_4703	beta-glucosidase [EC:3.2.1.21]	GH3
	QTS_4840	beta-glucosidase [EC:3.2.1.21]	GH3
	QTS_4897	glucosylceramidase [EC:3.2.1.45]	GH30
	QTS_4912	beta-galactosidase [EC:3.2.1.23]	
E. rectale	QTS_2580	oligo-1,6-glucosidase [EC:3.2.1.10]	GH13
	QTS_3119		GH53 + CBM16

The column “KEGG GH” shows the annotation conferred upon an OG by the KEGG GH database. The “HMM GH” shows the annotation of GH conserved domains (and adjacent CBM, if present) in an OG by the dbCAN HMMs.

Table S10. Locus tags of selected polysaccharide utilisation loci

**Xylan utilisation**					
**A.**					
CEn	RO1_29990	ROI_32860	RINT_01383	-	-
ABC-transporter component	RO1_30000	ROI_32870	RINT_01384	-	-
ABC-transporter component	RO1_30010	ROI_32880	RINT_01385	-	-
ABC-transporter component	RO1_30020	ROI_32890	RINT_01386	-	-
LacI-like transcriptional regulator	RO1_30040	ROI_32910	RINT_01388	-	-
Hypothetical protein	RO1_30050	ROI_32920	RINT_01389	-	-
GH51	RO1_30060	ROI_32930	RINT_01391	-	-
CE1	RO1_30070	ROI_32940	RINT_01392	-	-
CE1	RO1_30080	ROI_32950	RINT_01393	-	-
MATE-like efflux pump	RO1_30090	ROI_32960	RINT_01394	-	-
GH120	RO1_30100	ROI_32980	RINT_01396	-	-
GH51 + GH43 + CBM6	RO1_30110	ROI_32990	RINT_01397	-	-
GH39	RO1_30120	ROI_33000	RINT_01398	-	-
ABC-transporter component (ATPase)	-	ROI_33010	RINT_01400	-	-
Hypothetical protein	RO1_30300	ROI_33020	RINT_01401	-	-
GH43	-	-	-	-	T1_815_27531
AraC-like transcriptional regulator	RO1_30300	ROI_33040	RINT_01403c	M72_26321	T1_815_27511
GH39	RO1_30310	ROI_33050	RINT_01405	M72_26331	-
GH43	RO1_30320	ROI_33060	RINT_01406	M72_26341	T1_815_27501
GH51	RO1_30340	ROI_33080	RINT_01408	M72_26351	T1_815_27491
**B.**					
AraC-like transcriptional regulator	RO1_13560	ROI_23970	RINT_00879		
ABC-transporter system component	RO1_13570	ROI_23960	RINT_00880		
ABC-transporter system component	RO1_13590	ROI_23950	RINT_00881		
ABC-transporter system component	RO1_13600	ROI_23940	RINT_00882		
CE12	RO1_13610	ROI_23930	RINT_00883		
GH43	RO1_13620	ROI_23920	RINT_00884		
GH43	RO1_13630	ROI_23910	RINT_00885		
Hypothetical protein	RO1_13640	ROI_23900	RINT_00886		
GH28	RO1_13650	ROI_23890	RINT_00887		
Xylose isomerase	RO1_13660	ROI_23880	RINT_00888		
**Mucin utilisation**					
Mucin desulfatase	RINU_01915c	L1-83_03941			
GH112	RINU_01916c	L1-83_03951			
GH95	RINU_01918c	L1-83_03961			
GH95	RINU_01919c	-			
GH29	RINU_01921c	L1-83_03971			
ABC-transporter system component	RINU_01922c	L1-83_03981			
ABC-transporter system component	RINU_01923c	L1-83_03991			
ABC-transporter system component	RINU_01924c	L1-83_04001			
Response regulator containing CheY-like	RINU_01926	L1-83_04011			
Histidine kinase	RINU_01927	L1-83_04021			
Hypothetical protein	RINU_01928c	L1-83_04031			
Hypothetical protein	RINU_01929c	L1-83_04041			

**Blood group glycan utilisation**		
Hypothetical protein	RINU_02227c	
GH36	RINU_02228c	
GH109	RINU_02229c	
GH98 + 2 X CBM51	RINU_02230c	
GH29	RINU_02231c	
ABC-transporter system component	RINU_02232c	
ABC-transporter system component	RINU_02234c	
ABC-transporter system component	RINU_02235c	
Histidine kinase	RINU_02236c	
Response regulator containing CheY-like	RINU_02237c	
**Fructan utilisation**		
LacI-like transcriptional regulator	EUR_31790	EUBREC_0280
GH32 + CBM66	EUR_31800	EUBREC_0282
ABC-transporter system component	EUR_31810	EUBREC_0283
ABC-transporter system component	EUR_31820	EUBREC_0284
ABC-transporter system component	EUR_31830	EUBREC_0285
Hypothetical protein	EUR_31840	EUBREC_0286
LacI-like transcriptional regulator	ERE_03850	
ABC-transporter system component	ERE_03860	
ABC-transporter system component	ERE_03870	
ABC-transporter system component	ERE_03880	
GH32	ERE_03890	
Fructokinase	ERE_03900	
**Arabinogalactan utilisation**		
GH53 + CBM61	RHOM_10735	M72_25751
ABC-transporter system component	RHOM_10740	M72_25741
ABC-transporter system component	RHOM_10745	M72_25731
ABC-transporter system component	RHOM_10750	M72_25721
GH53	RHOM_10755	M72_25711
GH53 + CBM61	RHOM_10760	M72_25701
LacI-like transcriptional regulator	RHOM_10765	M72_25691
